# Shared Genetic Architecture Between Common Epilepsies and Subcortical Brain Volumes Is Associated With Cognition and Mental Health

**DOI:** 10.1002/cns.71156

**Published:** 2026-10-05

**Authors:** Zesheng Li, Qian Wu, Yanfeng Yang, Jianwei Shi, Xuegang Niu, Yuanyuan Zhang, Yumin Luo, Cheng Wei, Yongzhi Shan, Feng Liu, Josemir W. Sander, Guoguang Zhao

**Affiliations:** ^1^ Department of Neurosurgery Xuanwu Hospital, Capital Medical University Beijing China; ^2^ Clinical Research Center for Epilepsy Capital Medical University Beijing China; ^3^ China International Neuroscience Institute Beijing China; ^4^ Department of Radiology and Tianjin Key Laboratory of Functional Imaging & Tianjin Institute of Radiology Tianjin Medical University General Hospital Tianjin China; ^5^ Oncology and Pathology Karolinska Institutet Stockholm Sweden; ^6^ Department of Clinical and Experimental Epilepsy UCL Queen Square Institute of Neurology London UK; ^7^ Chalfont Centre for Epilepsy, Chalfont St Peter Buckinghamshire UK; ^8^ Neurology Department West China Hospital, Sichuan University Chengdu China

**Keywords:** comorbidities, genomic risk loci, GWAS, subcortical structure

## Abstract

**Aims:**

Epilepsy is a common neurological disorder, often presenting with psychiatric comorbidities and cognitive impairment. Neuroimaging studies have suggested associations between epilepsy and subcortical volumetric abnormalities. The genetic basis between epilepsy and subcortical volume abnormalities is, however, poorly understood, particularly if they share a common genetic architecture.

**Methods:**

To investigate polygenic overlap, we employed the bivariate causal mixture model (MiXeR) to analyze genome‐wide association data from various epilepsy subtypes (genetic generalized epilepsy [GGE]: *n* = 49,388, all epilepsy [EP]: *n* = 69,995, focal epilepsy: *n* = 57,375) and 16 subcortical volumetric phenotypes (*n* = 33,224). We identified shared genomic loci through conditional and conjunctional false discovery rate analyses (cond/conjFDR).

**Results:**

MiXeR results suggest substantial shared genetic variants between subcortical volume and common epilepsies. ConjFDR analysis identified 33 distinct shared loci between GGE and subcortical volumetric phenotypes. The shared loci were mapped to 322 protein‐coding genes. Among these, 49 genes showed significant expression‐trait associations with both GGE and subcortical volume phenotypes, and 12 of the 322 genes were also associated with cognitive, mental health, and personality‐related traits.

**Conclusion:**

We dissected the shared genetic architecture between common epilepsies and subcortical volumes. Expression‐trait association analyses further identified candidate genes associated with cognitive, mental health, and personality‐related phenotypes.

## Graphical abstract

Epilepsy is a common neurological disorder, often presenting with psychiatric comorbidities and cognitive impairment. Neuroimaging studies have suggested associations between epilepsy and subcortical volumetric abnormalities. Here, we dissected the shared genetic architecture between common epilepsies and subcortical volumes. These loci are associated with behaviors.

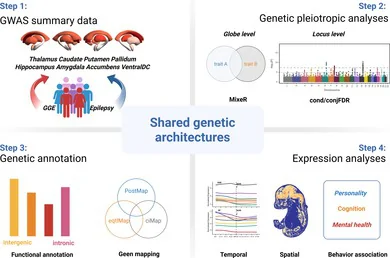

## 1 Introduction

Epilepsy is a neurological disorder affecting over 70 million individuals worldwide [1]. The condition is characterized by recurrent, unprovoked epileptic seizures, which impact quality of life [2]. Significant psychosocial comorbidities, including anxiety, depression, cognitive impairment, and personality change, often increase the burden of epilepsy [3, 4, 5, 6]. Subcortical brain structures, such as the amygdala and hippocampus, play a significant regulatory role in domains such as cognition, mental health, and personality [7, 8, 9, 10, 11, 12]. Uncovering overlapping genetic architecture between epilepsy and subcortical brain structures may facilitate a better understanding of epilepsy pathophysiology and related comorbidities.

Neuroimaging studies have shown that subcortical volume abnormalities are present across different epilepsy syndromes. Epilepsy is stratified into four major subtypes: genetic generalized epilepsy (GGE), focal epilepsy (FE), unclassified, and unknown whether focal or generalized epilepsy [2]. GGE represents epilepsy syndromes characterized by generalized seizure activity. People with GGE show volume changes in the thalamus, hippocampus, putamen, and globus pallidus [13, 14, 15]. In FE, seizure onset is localized in a specific brain area. Volume differences in subcortical structures (thalamus, hippocampus, putamen, amygdala, and caudate) are frequently observed in people with FE compared to those without epilepsy [4, 16, 17, 18, 19, 20, 21]. Subcortical abnormalities, in some, are already detectable at the onset of epilepsy [22], implying that these changes likely originate from early pathological processes rather than from chronic seizures or antiepileptic treatment [23]. Investigating the shared genetic architecture between common epilepsies and subcortical brain volumes is crucial for understanding the phenomena.

Abnormal subcortical volumes are also linked to cognitive deficits, psychiatric symptoms, and personality changes in people with epilepsy [16, 19, 20, 23, 24]. Since people with epilepsy often show differences in subcortical volumes, and these volumes are associated with cognition, mental health, and personality traits, we speculate that there may be shared genetic architecture between epilepsy and subcortical structures that underlie cognition, mental health, and personality changes.

Epilepsy with genetic contributions varying across different subtypes highlights the importance of genetic studies [25, 26, 27, 28, 29, 30]. While genome‐wide association studies (GWAS) have identified genetic variants associated with brain structure and epilepsy phenotypes, the extent of genetic overlap between epilepsy and subcortical brain structures remains insufficiently explored. A recent study using linkage disequilibrium score regression (LDSC) analysis revealed genetic correlations between the globus pallidus and GGE [31]. This study did not find a correlation between other subcortical structures and GGE. The LDSC method, however, tends to underestimate the extent of genetic sharing, especially when genetic variants have a mix of concordant and discordant effects on the traits. A bivariate causal mixture model (MiXeR), which estimates genetic overlap by including all variants, regardless of their effect directions, has been proposed [32]. Additionally, conditional and conjunctional false discovery rate (cond/conjFDR) methods employ an empirical Bayesian framework to increase the discovery potential of GWASs for novel risk loci and loci shared between two traits [33]. The use of these methods has substantially advanced our understanding of the shared genetic architecture across complex traits [34, 35].

In this study, we used MiXeR and condFDR/conjFDR methods to assess the shared genetic architecture and novel loci between common epilepsies and 16 subcortical volumes. We aimed to advance our understanding of the shared mechanisms between common epilepsies and subcortical volumes, as well as their association with cognition, mental health, and personality.

## 2 Materials and Methods

### 2.1 GWAS Data

We used GWAS data from individuals of European ancestry. Summary epilepsy GWAS data were sourced from the Epilepsy Genetic Association Database (epiGAD) [25]. The sample includes 69,995 subjects, defined as EP (27,559 cases, 42,436 controls). The dataset is focused on three major epilepsy phenotypes: GGE (6952 cases, 42,436 controls), FE (14,939 cases, 42,436 controls), and unclassified epilepsy. Additionally, summary statistics for 16 subcortical volumetric phenotypes, encompassing bilateral volumes of the thalamus, caudate, putamen, pallidum, hippocampus, amygdala, accumbens, and ventral diencephalon (ventralDC), were obtained from the UK Biobank, comprising a sample size of 33,224 individuals [36].

### 2.2 Univariate and Bivariate MiXeR Analyses

We utilized the MiXeR (v1.3) statistical tool to quantitatively evaluate the polygenic overlap between common epilepsies and subcortical volumetric traits [32, 37]. Univariate MiXeR analyses were conducted to assess heritability, polygenicity, and discoverability for each phenotype [37]. Before fitting bivariate models, we observed positive Akaike information criterion (AIC) values for all traits except FE, supporting sufficient statistical power for bivariate MiXeR analyses of the remaining traits. Bivariate MiXeR analyses were then conducted to model the estimated additive genetic effects using a mixture of four Gaussian components [32]. For a detailed description of MiXeR, see Appendix S1.

### 2.3 Cond/conjFDR Analyses

To improve genetic variant discovery for individual traits, we applied the condFDR statistical framework to re‐rank the test statistics of the primary phenotype [33]. This re‐ranking was based on the strength of their association with a secondary phenotype. We excluded SNPs around the MHC (GRCh37/hg19: chr6: 25000000–35000000) and 8p23.1 (GRCh37/hg19: chr8: 7200000–12500000) region before fitting the model to avoid bias due to their complex regional LD [34, 35]. Then we conducted a conjFDR analysis to identify genomic loci jointly associated with both traits. The significance thresholds were set at 0.01 for condFDR and 0.05 for conjFDR [34, 35]. For a detailed description of how the novelty of loci identified by cond/conjFDR was determined, see Tables S1 and S2 and Appendix S1 [6, 10, 25, 29, 31, 38, 39, 40, 41, 42, 43, 44, 45, 46, 47].

### 2.4 Spatial Mapping Analysis

We applied gsMap to chart the spatial distribution and organ specificity of genes shared between GGE and subcortical brain structures, focusing exclusively on genes with statistically significant expression‐trait associations in GGE and at least one subcortical region. The analysis proceeded in two steps: (1) learning latent representations for spots; (2) generating gene specificity scores (GSS) for each spot by aggregating information from homogeneous spots [48].

### 2.5 Cognitive, Mental, and Personality Associations

We analyzed GWAS summary data covering eight cognitive domains [49], two core mental health disorders and five personality dimensions [50, 51, 52, 53, 54]. The goal was to assess whether genes shared between GGE and subcortical brain structures are linked to common behavioral features observed in people with epilepsy. We applied the “S‐MultiXcan” method to test for associations between the expression levels of shared genes and cognitive, mental, and personality traits. Associations with Benjamini–Hochberg FDR‐adjusted *q* < 0.05 were considered significant.

## 3 Results

### 3.1 Investigation of Polygenic Overlap With MiXeR

The workflow of this study was shown in Figure 1. The univariate models showed a satisfactory fit for all subcortical volumetric phenotypes and GGE, EP phenotypes, as indicated by positive AIC values. As shown in Figure 2A and Table S3, our analysis revealed significant differences in the genetic architecture of epilepsy phenotypes and subcortical brain volumes. The SNP heritability for epilepsy ranged from 0.077 in EP to 0.475 in GGE, with GGE exhibiting the highest SNP heritability among all the phenotypes studied. In contrast, subcortical volumes showed moderate heritability, ranging from 0.189 to 0.309, with the highest heritability observed in the left caudate. Epilepsy phenotypes exhibited relatively higher polygenicity, with the number of trait‐influencing SNPs ranging from 2279 to 2904, peaking in the EP phenotype. In comparison, subcortical volumes displayed a broader range of SNP influences, with 1110 to 3213 variants identified. Discoverability estimates for epilepsy phenotypes ranged from 4.16 × 10^−5^ to 2.54 × 10^−4^, with GGE demonstrating the highest discoverability (2.54 × 10^−4^). Conversely, subcortical volumes showed higher discoverability, ranging from 1.19 × 10^−4^ to 3.13 × 10^−4^. Bilateral hippocampal volumes exhibited particularly elevated discoverability estimates, with the left hippocampus reaching the highest value (3.13 × 10^−4^) across all phenotypes examined.

**FIGURE 1:**
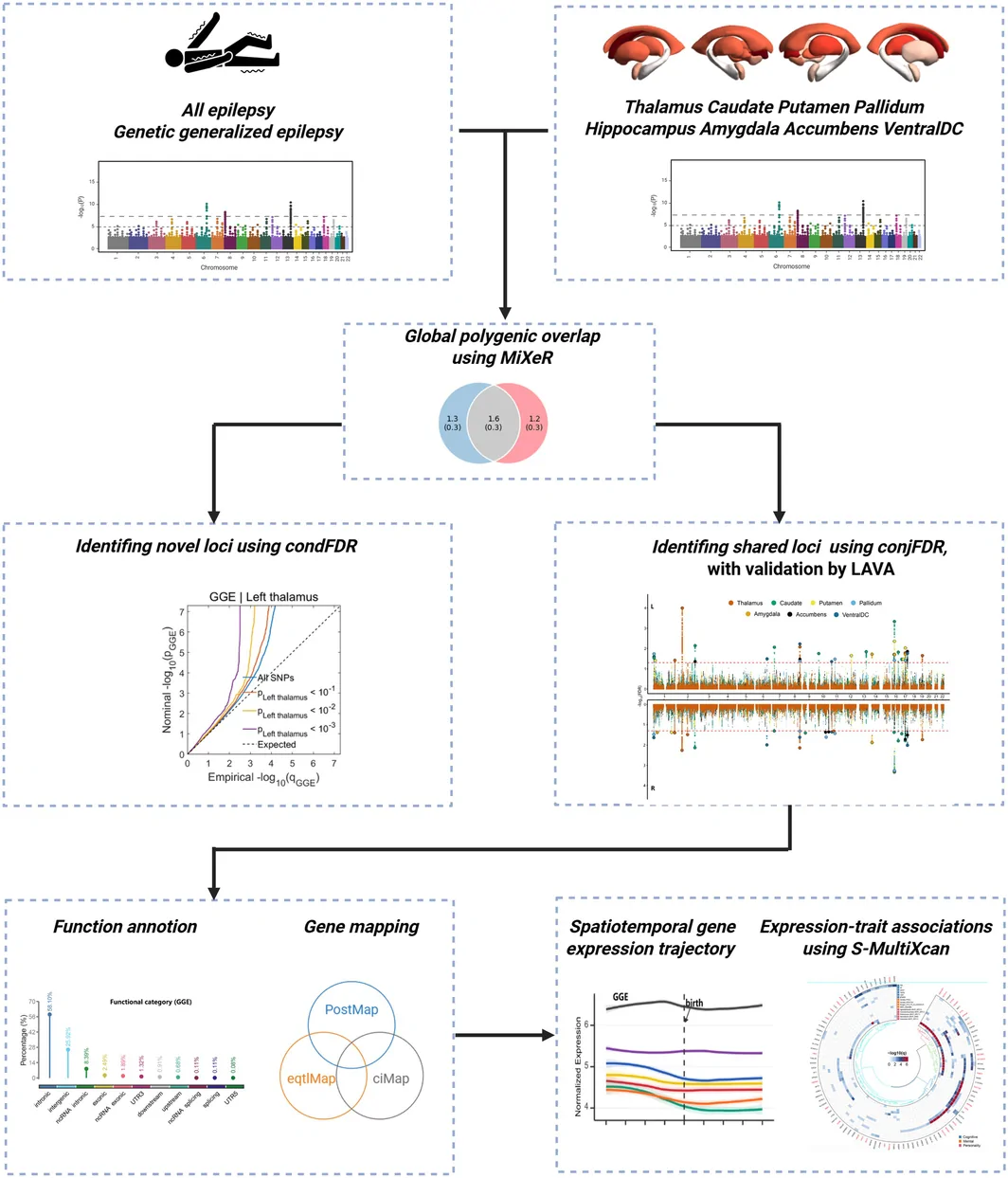
Workflow of this study.

**FIGURE 2:**
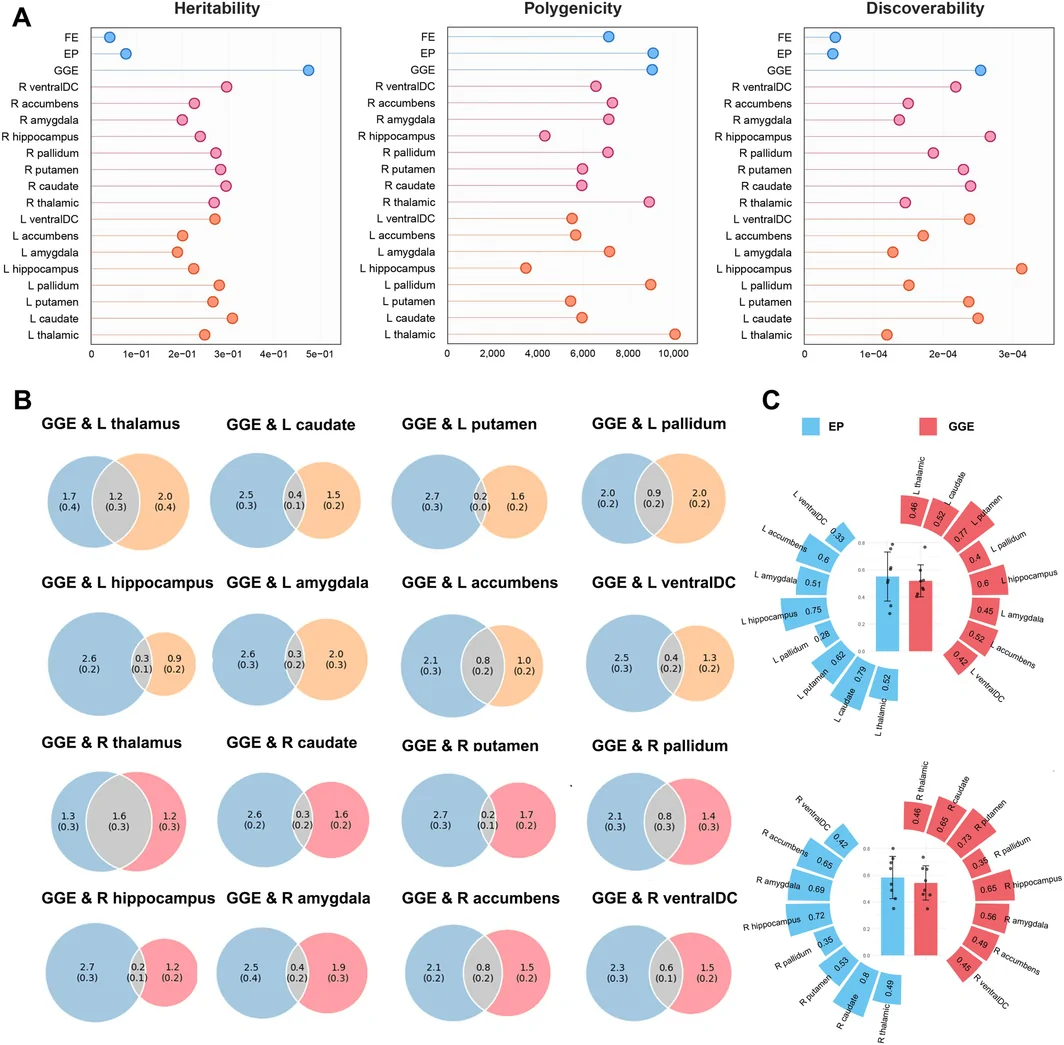
Genetic overlap between epilepsy and subcortical brain volumes using MiXeR. (A) Heritability, polygenicity, and discoverability for each phenotype estimated by univariate MiXeR. (B) Venn diagrams showing MiXeR results of the estimated number of variants shared between genetic generalized epilepsy (GGE) and subcortical brain volumes. The numbers of shared and trait‐specific variants are shown in thousands with standard errors. The size of the circles reflects the degree of polygenicity. (C) The outer ring displays the proportions of shared variants with concordant effect directions between each epilepsy subtype (all epilepsy and GGE) and subcortical brain volumes. The central bar plot shows the overall proportion of such variants for the two types of epilepsy (blue: all epilepsy, red: GGE). L, left; R, right.

Through bivariate MiXeR analysis, we identified substantial shared genetic variants between epilepsy and subcortical volumetric phenotypes (Tables S4 and S5, Figure 2B, Figures S1–S33). Due to the negative AIC value and insufficient power in the fit of the bivariate MiXeR model, the results of the bivariate MiXeR analysis in FE were excluded (Table S3, Figures S34–S49). As shown in Figure 2B, Figure S1, and Tables S4 and S5, GGE exhibited a proportion of shared trait‐influencing variants with subcortical structures (44.0%–94.5%) and EP exhibited 55.5%–89.6%. Conversely, subcortical volumetric traits shared 43.1%–90.8% of their trait‐influencing variants with GGE and 42.6%–84.8% with EP. The Dice coefficient (GGE: 6.9%–56.4%; EP: 13.0%–44.3%), representing the bidirectional genetic overlap between trait pairs, revealed distinct sharing patterns across epilepsy‐subcortical volume combinations.

The proportion of shared variants with concordant allelic effects between each epilepsy phenotype and subcortical volume ranged from 34.9% to 76.7% for GGE and 27.7% to 80.0% for EP (Figure 2C, Tables S4 and S5). Within the shared component, for GGE, positive correlations were found with the bilateral caudate, putamen, hippocampus, left accumbens, and right amygdala, with the maximum correlation (rho = 0.725) observed in the left caudate. In contrast, GGE exhibited negative correlations with all other subcortical structures, with the minimum correlation (rho = −0.452) in the right pallidum, as shown in Table S4. For EP, positive correlations were identified with the left thalamus and bilateral caudate, putamen, hippocampus, amygdala, and accumbens, with the highest correlation (rho = 0.783) in the right caudate. EP also showed negative correlations with all other subcortical structures, with the lowest correlation (rho = −0.619) observed in the left pallidum, as shown in Table S5. All epilepsy phenotypes consistently exhibited negative correlations with the pallidum.

### 3.2 Identification of Novel and Shared Genetic Loci

Using a condFDR threshold of < 0.01 and conditioning on subcortical volumetric phenotypes, we identified risk loci for common epilepsies. Specifically, we identified 625 genomic risk loci associated with GGE, and when combined with all subcortical phenotypes, these merged into 68 distinct loci, of which 18 were novel (Tables S6 and S7, Figure S50). For EP, 141 loci were detected overall, yielding 29 distinct loci, and 12 of them were novel (Tables S8 and S9, Figure S51).

When conditioning on epilepsy phenotypes, we identified loci associated with subcortical volumetric traits. Specifically, conditioning on GGE revealed 508 loci associated with subcortical volumetric phenotypes. These merged into 250 distinct loci when combining all subcortical volumetric traits, with nine of them novel (Tables S10 and S11, Figure S52). Similarly, when conditioning on EP, we identified 466 loci associated with subcortical volumetric phenotypes, which aggregated to 231 distinct loci, including seven newly identified regions (Tables S12 and S13, Figure S53). The number of loci identified by condFDR analysis was shown in Figure S54. Conditional quantile‐quantile (*Q–Q*) plots highlighted the genetic overlap for GGE‐volume pair and EP‐volume pair, while indicating that the FE was underpowered (Figures S55–S102). The FE‐volume pair was excluded from cond/conjFDR analyses.

Then, conjFDR analysis was used to find genomic loci jointly associated with both traits. Using a conjFDR threshold of < 0.05, we identified genetic loci jointly associated with epilepsy phenotypes and subcortical brain volumes. For GGE, we discovered 68 genetic loci shared with subcortical brain volumes, distributed across thalamus, caudate, putamen, pallidum, ramygdala, accumbens, and ventralDC, with no associations in the hippocampus. After consolidating overlapping genomic regions, these 68 loci were merged into 33 distinct shared loci across all GGE‐volume pairs. Among these shared loci, 19 were novel for GGE, seven were novel for subcortical volumetric phenotypes, and three were novel for both phenotypes (Figure 3A, Tables S14 and S15).

**FIGURE 3:**
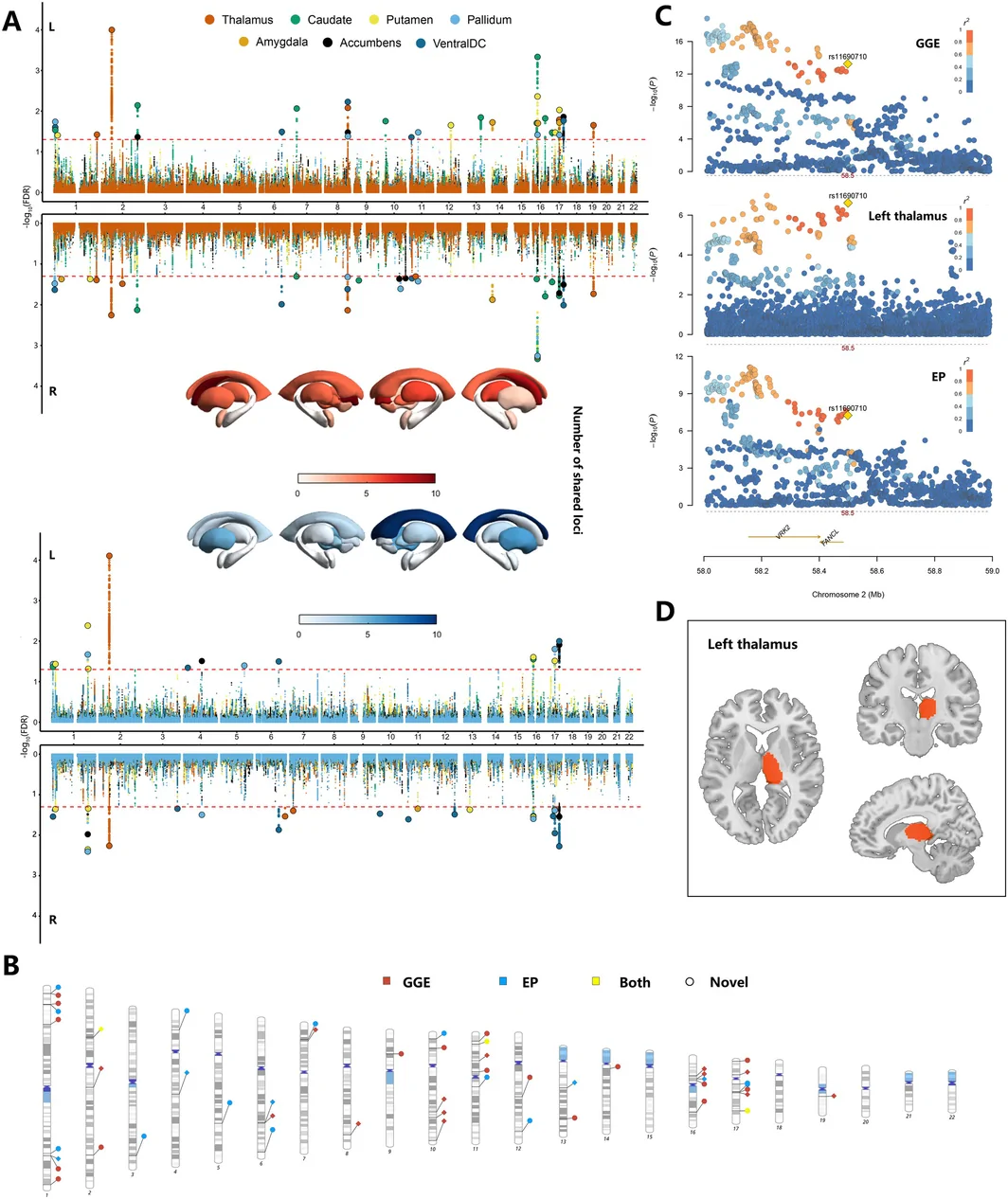
Shared loci between epilepsy and subcortical brain volumes. (A) The upper Manhattan plot illustrates the associations between genetic generalized epilepsy (GGE) and subcortical brain volumes identified by conjFDR analysis, while the lower Manhattan plot depicts the associations for all epilepsy (EP) and subcortical brain volumes. Each point represents a SNP, with larger points indicating lead SNPs. The *y*‐axis displays the −log_10_‐transformed conjFDR values for each SNP, and the *x*‐axis denotes chromosomal positions. The dashed line marks the significance threshold of conjFDR = 0.05, and point colors correspond to different phenotypes. The brain map illustrates the number of genetic loci within each subcortical structure that are shared with GGE (in red) and with EP (in blue). (B) Distribution of 33 distinct loci shared between GGE and subcortical brain volumes, 21 distinct loci shared between EP and subcortical brain volumes, and three distinct loci shared between GGE‐volume pairs and EP‐volume pairs by conjFDR analyses, spanning across the genomic landscape of chromosomes. Circular dots indicate that the loci are novel for GGE or EP. (C) LocusZoom plots of the genomic region at chr2: 57942325–58505679 (lead SNP: rs11690710) display the regional SNP associations, represented as two‐sided −log_10_(*p*) values for GGE, left thalamus volume, and EP based on original GWAS summary statistics. (D) MRI slice plot of left thalamus volumes. L, left; R, right.

For EP, we identified 44 genetic loci shared with subcortical brain volumes, distributed across thalamus, caudate, putamen, pallidum, right amygdala, accumbens, and ventralDC, with no associations in hippocampus or left amygdala. These 44 loci merged into 21 distinct shared loci, of which 15 were novel for EP, 4 were novel for subcortical volumetric phenotypes, and two were novel for both (Figure 3A, Tables S16 and S17). Three distinct loci shared between the GGE‐volume and EP‐volume pairs were identified by conjFDR analysis (Figure 3B).

Among the identified shared loci of GGE‐volumes pair and EP‐volumes pair, chr2: 57942325–58505679 (lead SNP: rs11690710) emerged as the most significant, showing strong pleiotropic associations between GGE and left thalamus volume (conjFDR = 1.01E‐04), as well as between EP and left thalamus volume (conjFDR = 7.80E‐05) (Figure 3C,D, Tables S15 and S17).

### 3.3 LAVA Validation

Results from LAVA consistently corroborated our conjFDR findings across the GGE and EP epilepsy subtypes and their relationships with subcortical brain volumes. For the 68 shared loci between GGE and subcortical brain volumes, LAVA identified 55 genetic loci (80.9%) with nominally significant (*p* < 0.05) local heritability in both phenotypes. Among these, 30 loci showed significant local genetic correlation (FDR *q* < 0.05). These loci displayed bidirectional effects, with 17 loci showing positive correlation (ranging from 0.82 to 1.00) and 13 exhibiting negative correlation (ranging from −1.00 to −0.78). Similarly, for the 44 shared loci between EP and subcortical volumes, LAVA confirmed 31 loci (70.5%) with significant local heritability in both phenotypes. Of these, 19 demonstrated significant genetic correlation (FDR *q* < 0.05), with 10 showing positive correlation (ranging from 0.70 to 1.00) and nine displaying negative correlation (ranging from −1.00 to −0.94). Complete correlation statistics for all shared loci are presented in Tables S14 and S16.

### 3.4 Functional Annotation

Functional annotations of candidate SNPs revealed similar genomic distributions across epilepsy subtypes, with the majority located in intronic (GGE: 58.10%; EP: 65.94%) and intergenic (GGE: 25.92%; EP: 22.76%) regions, as illustrated in Figure 4A,B and detailed in Tables S18 and S19. Additionally, among candidate SNPs shared between epilepsy subtypes and subcortical volumes, we identified 15 non‐synonymous exonic variants for GGE compared to only one for EP. Both epilepsy types showed comparable proportions of potentially pathogenic variants (CADD scores > 12.37: GGE 5.20%, EP 5.08%) and regulatory variants (RegulomeDB score < 3: GGE 5.91%, EP 5.78%). Additionally, most candidate SNPs were located within open chromatin regions (chromatin state score < 8: GGE 90.87%, EP 87.04%). Gene mapping using three‐way strategies linked the candidate SNPs in distinct shared loci between GGE and subcortical brain volumes to 322 protein‐coding genes, yielding significant enrichment for 19 GO biological processes (FDR *q* < 0.05) (Tables S20 and S21). The most significant terms included positive regulation of cellular process (FDR *q* = 7.79E‐04) and positive regulation of biological process (FDR *q* = 7.79E‐04) (Figure 4C). In contrast, the candidate SNPs in distinct shared loci between EP and subcortical brain volumes mapped to 159 protein‐coding genes, yielding significant enrichment for five GO biological processes (Tables S22 and S23). The significant terms included cell cycle (FDR *q* = 1.47E‐02) and positive regulation of metabolic process (FDR *q* = 3.02E‐02) (Figure S103).

**FIGURE 4:**
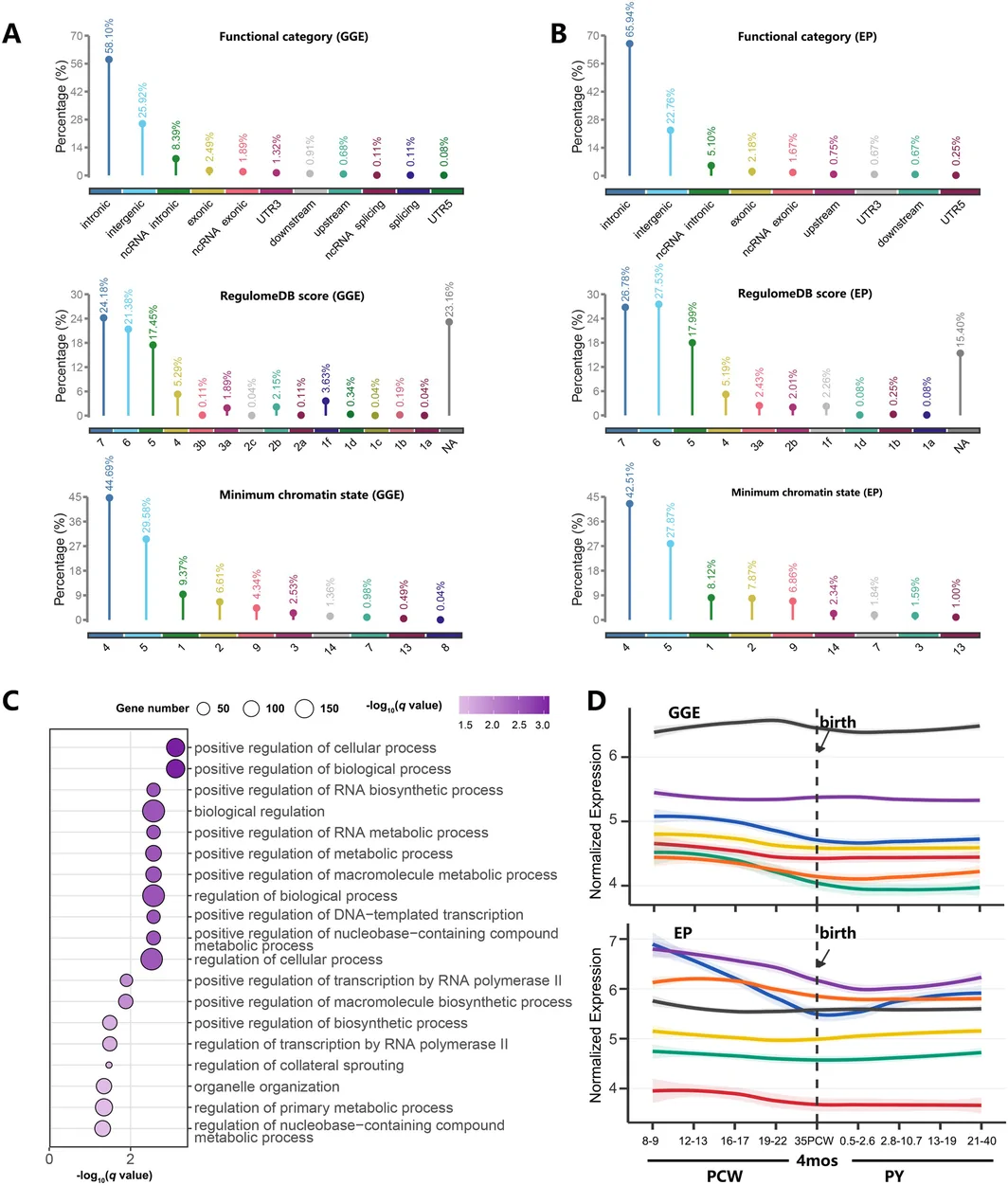
Functional annotation for the shared loci. (A) Distribution of functional categories, RegulomeDB scores, and minimum chromatin states of candidate SNPs in the genomic loci shared between genetic generalized epilepsy (GGE) and subcortical volumes. (B) Distribution of functional categories, RegulomeDB scores, and minimum chromatin states of candidate SNPs in the genomic loci shared between all epilepsy (EP) and subcortical volumes. (C) This bubble plot illustrates the results of Gene Ontology biological process enrichment analysis for shared genes. The horizontal axis represents −log_10_‐transformed values of FDR‐corrected two‐sided *p* values, while the vertical axis displays individual Gene Ontology terms. The color intensity of each marker indicates the magnitude of −log_10_(*q*) values, and marker size corresponds to the number of genes associated with each functional category. (D) Gene expression trajectories (PsychENCODE) for genes mapped from shared candidate SNPs (conjFDR < 0.05) between GGE (top)/EP (bottom) and subcortical volumetric phenotypes (left and right subcortical phenotypes are combined). ncRNA, noncoding RNA; PCW, post‐conceptional week; PY, postnatal year.

We conducted gene mapping of the novel loci identified through cond/conjFDR analyses using three‐way strategies (Tables S24–S31). To explore the biological processes associated with these novel loci, we performed GO biological process enrichment analysis on the mapped genes. The genes mapped to the novel GGE loci are involved in regulation of cellular and metabolic processes (Table S32, Figure S104). The genes mapped to the novel EP loci are involved in nuclear division, the cell cycle, and molecule localization‐related biological processes (Table S33, Figure S105). The genes mapped to the novel loci in subcortical structures showed no significant functional enrichment.

### 3.5 Developmental Trajectories of Shared Genes

Utilizing spatiotemporal brain expression trajectory data from PsychENCODE, we explored the developmental patterns of genes shared between epilepsy subtypes and each subcortical structure throughout the lifespan. As depicted in Figure 4D, GGE and EP exhibited similar overall developmental expression trajectories across brain regions, yet with notable subtype‐specific differences. For genes shared between GGE and subcortical structures, distinct developmental trajectories were observed across brain regions. The ventralDC and accumbens consistently maintained high expression levels throughout development, demonstrating stability from the prenatal to the adult stages. The thalamus exhibited continuous decline from early prenatal phases through the perinatal transition, subsequently stabilizing postnatally. The caudate, putamen, and amygdala all displayed downward trajectories from prenatal to postnatal periods, with the putamen showing the most pronounced decline. The pallidum remained relatively stable across developmental windows with minor fluctuations.

In contrast, genes shared between EP and subcortical structures demonstrated more dynamic perinatal transitions. The accumbens and thalamus exhibited characteristic U‐shaped trajectories, with high expression during early prenatal stages (Windows 1–4), reaching minimum levels at the perinatal period (Window 5), followed by gradual recovery postnatally. The putamen, pallidum, and ventralDC maintained relatively stable expression patterns throughout development. The caudate and amygdala showed gradual declining trends that stabilized after birth (Figure 4D).

### 3.6 Expression‐Trait Associations

Given that shared genes between EP and subcortical volumes weakly enrich significant biological functions, subsequent expression trait analyses were conducted for shared genes between GGE and subcortical volumes. For each GGE‐volume pair, we investigated whether the expression of shared genes in the brain was associated with GGE and the corresponding subcortical volumes using the “S‐MultiXcan” tool. Out of these shared genes, we found 187 significant associations between gene expression (involving 96 genes) and volumetric phenotypes. Among these 96 genes, 49 genes were also found to be associated with GGE (Tables S34 and S35, Figure 5A). Specifically, the brain expression levels of *TUFM*, *SULT1A1*, *NPIPB9*, *SULT1A2*, *EIF3CL*, and *NPIPB7* were significantly associated with bilateral caudate and putamen volumes as well as GGE. Among the 49 genes, *VRK2* exhibited the most significant expression‐GGE association and was concurrently linked to bilateral thalamic volumes. Consistent with the conjFDR analysis results, no shared genes were identified whose expression was simultaneously associated with GGE and bilateral hippocampal volumes. The shared genes exhibited clear lateralisation, with more shared genes simultaneously linked to GGE and left putamen and amygdala volumes than to GGE and right putamen and amygdala volumes.

**FIGURE 5:**
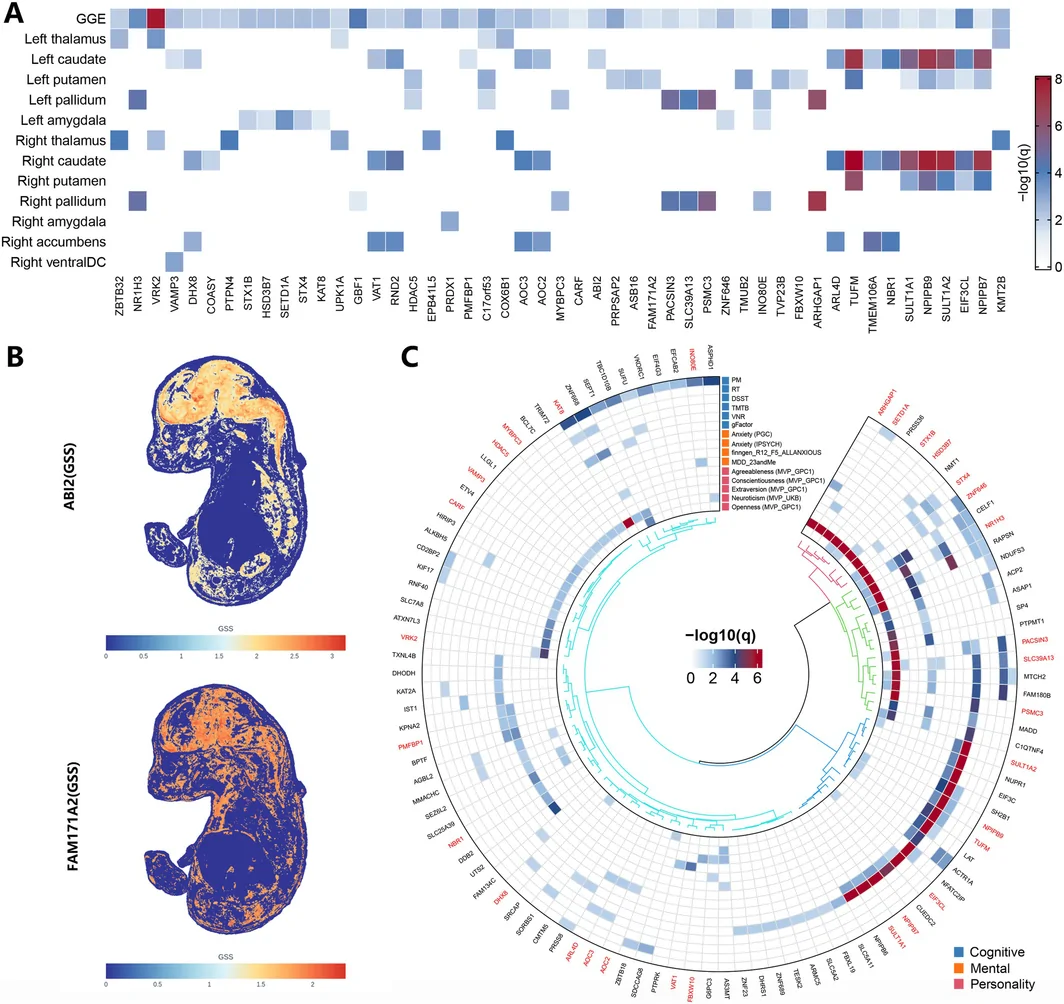
Associations of the expression of shared genes between genetic generalized epilepsy (GGE) and subcortical brain volume in the brain with cognitive abilities, mental health, and personality. (A) The heatmap displays all S‐MultiXcan‐identified expression‐trait associations (*q* < 0.05, FDR‐corrected) for genetic generalized epilepsy (GGE) and at least one subcortical brain volume. Forty‐nine genes are shown on the *x*‐axis, with GGE and subcortical structures on the *y*‐axis. Association strengths (−log_10_(*q*)) are visualized by a white‐to‐red color scale. (B) Spatial distributions of GSS for *ABI2* and *FAM171A2*. The color scale represents GSS values, with blue indicating low specificity and red indicating high specificity. (C) Heatmap showing significant expression‐trait associations (*q* < 0.05, FDR corrected) between shared genes for GGE and subcortical brain volumes (*x*‐axis) and cognitive, mental, and personality phenotypes (*y*‐axis). Color intensity represents association significance (−log_10_(*q*)), with white indicating nonsignificant results.

We also conducted spatial mapping analysis of 49 shared genes associated with GGE and at least one volumetric phenotype. Our findings demonstrated that *ABI2* and *FAM171A2* presented brain‐specific enrichment as early as E16.5 embryonic tissue (Table S36, Figure 5B). As illustrated in Figure 5A, *ABI2* is associated with GGE and the left caudate. In contrast, *FAM171A2* is associated with GGE and the left putamen.

### 3.7 Cognitive, Mental, and Personality Associations

We analyzed GWAS summary data encompassing eight cognitive abilities, two psychiatric disorders (anxiety and depression; four GWAS datasets), and five personality traits (Table S37). The associations between the expression of 322 shared genes and cognitive, mental, and personality phenotypes were further examined using “S‐MultiXcan.” The results showed that 101 genes were associated with at least one clinical phenotype (Table S38). Among them, 31 genes showed significant associations between their expression levels in brain tissue and GGE, as well as at least one subcortical volume. Among the 101 genes, 64 genes were associated with cognitive phenotypes, 34 genes with mental health‐related phenotypes, and 58 genes with personality phenotypes. Twelve genes simultaneously showed associations with three phenotype categories (cognitive, mental, and personality), including *CELF1*, *SLC39A13*, *NR1H3*, *MTCH2*, *MADD*, *ACP2*, *ASAP1*, *RAPSN*, *AGBL2*, *C1QTNF4*, *FAM180B*, and *NDUFS3* (Table S38, Figure 5C). Among these 12 genes, *SLC39A13* and *NR1H3* were supported in the former expression‐trait associations analysis, confirming the associations between their brain tissue expression levels and GGE. Interestingly, the brain expression of *SLC39A13* and *NR1H3* showed common associations with bilateral pallidum volume and GGE (Tables S34 and S35, Figure 5A).

## 4 Discussion

We revealed the shared genetic architecture between common epilepsies and subcortical brain volumes. Over one‐third of these shared variants exerted opposing effects on the two traits, consistent with the concept of antagonistic pleiotropy. This mixture of concordant and discordant effects explains the previously detected weak genome‐wide genetic correlation between GGE and subcortical brain volumes [31]. The cross‐trait strategy reveals novel and shared genetic loci that are undetected in single‐trait analyses.

Shared loci can illuminate the pathophysiology underlying the comorbidity of epilepsy and structural brain differences. Among the shared loci between the GGE‐volumes pair and EP‐volumes pair, one novel locus for both GGE and EP is located on chr17: 65824046–66096451, intronic within *BPTF* gene, and the lead SNP is rs1976054. Previous studies have demonstrated that *BPTF* haploinsufficiency is associated with a neurodevelopmental disorder characterized by dysmorphic facies and distal limb anomalies (NEDDFL). A subset of these people had epileptic seizures, leading to the recent classification of BPTF‐related epilepsy [55]. In previous studies, BPTF‐related epilepsy did not demonstrate genotype–phenotype correlation. This is mainly attributed to the fact that all identified variants were of the loss‐of‐function type, which could result in protein truncation and subsequent nonsense‐mediated decay [55, 56]. This poses a challenge for understanding why epileptic seizures occur in some individuals affected by NEDDFL. Using cond/conjFDR analyses, the novel *BPTF* gene locus was found to be associated with GGE, EP, and subcortical volumes. These results offer GWAS evidence supporting the relationship between *BPTF* genotype and phenotype in GGE and EP. It also highlights the need to explore potential differences in subcortical brain structures among individuals with BPTF‐related neurodevelopmental disorders.

In the conjFDR analyses, the null findings for hippocampal volume in relation to GGE and EP require cautious biological interpretation. First, the imaging GWAS captures volumetric variation in a general population rather than in a clinical epilepsy cohort. Second, GGE is more strongly linked to thalamocortical circuitry than to hippocampal pathology. Third, the absence of FE‐specific analyses may have limited our ability to detect hippocampus‐related genetic signals.

Cellular metabolism is emerging as a common biological framework within epilepsy. The shared genes in the GGE‐volumes pair and EP‐volumes pair are all enriched in metabolic processes. Spontaneous recurrent seizures can induce secondary aberrations in cellular metabolism [57]. Inherent defects in brain energy metabolism can also trigger seizures. Mutations in genes that encode mitochondrial proteins, metabolic substrates, or enzymes, as well as dysfunctions in epigenetic processes, all play roles in this aspect [57]. The metabolic, biochemical, and epigenetic transcriptional alterations have important roles in the pathophysiology of epilepsy, and there is a connection among them. For example, seizures can lead to the release of adenosine, which serves as a component of RNA and regulates the S‐adenosylmethionine‐dependent transmethylation pathway [58, 59]. Epileptic seizures indicate a state of excessive energy consumption. This energy crisis can result in decreased ATP levels, which subsequently impairs RNA synthesis while also elevating adenosine levels that influence DNA methylation [57]. This aligns with the biological processes we identified (Figure 4C).

Conventional pharmacotherapy development typically begins with druggable targets, such as G protein‐coupled receptors, ion channels, and protein kinases. However, alterations in metabolism‐related RNA, peptides, and proteins, which ultimately lead to epigenetic changes, may underlie the abnormalities associated with these targets [57]. By emphasizing the metabolic basis of epilepsy, new therapeutic avenues may be discovered.

Among the 12 genes associated with cognitive, mental health, and personality traits, *SLC39A13* and *NR1H3* were significantly associated with bilateral pallidum volume as well as with cognitive, mental health, and personality traits. We therefore focus on the function of the two genes. *SLC39A13* is a zinc transporter, and its rare mutations lead to comprehensive cognitive dysfunction, severe psychopathology, and the lowest levels of intelligence and educational attainment [60, 61]. Animal models confirm this, as gene knockout induces depression‐like behaviors that are improved by zinc supplementation [62]. *NR1H3* is a transcription factor that inhibits inflammation, with its variants linked to autoimmune and neuroinflammatory diseases, including anti‐NMDAR encephalitis, multiple sclerosis, and systemic lupus erythematosus [63, 64, 65, 66]. This suggests them as high‐priority targets for molecular interventions designed to address GGE and its neuropsychiatric comorbidities.

Our study has limitations. Firstly, the analysis relies on GWAS data derived from populations of European ancestry. Continued exclusion of diverse ancestries risks an inequitable distribution of the benefits of genetic research and could exacerbate existing health disparities [67]. Future work should seek to replicate these findings in samples with greater ancestral diversity. Secondly, large datasets can markedly increase the statistical power of genomic analyses; however, they remain limited in characterizing environmental exposures and longitudinal disease trajectories. Future longitudinal follow‐up studies are needed. This will map how epilepsy and subcortical brain structures change over time and clarify how genetic and socio‐environmental factors interact. Thirdly, the conjFDR method employed faces inherent challenges in directly identifying true causal variants. These challenges arise because linkage disequilibrium may result in multiple pathogenic variants underlying overlapping loci. We functionally annotated the candidate SNPs with the highest potential to be causal variants. However, experimental validation of these variants remains essential for confirming causality. Our analyses focused exclusively on common autosomal SNPs, without addressing rare variants or sex chromosome variations, which may also contribute significantly to disease pathogenesis and should be incorporated in subsequent studies. The UK Biobank participants were not subject to exclusion of individuals with epilepsy or cognitive impairment. Nevertheless, because the analyses were based on population‐level GWAS summary statistics and the imaging phenotypes were adjusted for multiple relevant confounders, this issue is unlikely to substantially alter the overall genetic‐overlap findings. The study uses GWAS of subcortical brain volumes and GWAS of cognitive, psychiatric, and personality traits, but does not analyze individual‐level data from people with epilepsy with direct measurements of cognition, mental health, or personality. Therefore, the results cannot directly demonstrate that the identified genes explain comorbidities in people with epilepsy. Lastly, the bivariate MiXeR statistical methodology is limited to two‐variable analyses, which makes it difficult to control for other potential confounding factors. Future research with larger sample sizes, more diverse populations, and more comprehensive datasets is needed to validate these findings and further refine our understanding of the genetic mechanism of the disease.

## Author Contributions

**Zesheng Li:** formal analysis, visualization, writing – original draft. **Qian Wu:** validation, software. **Yanfeng Yang:** data curation, validation. **Jianwei Shi:** resources. **Xuegang Niu:** investigation. **Yuanyuan Zhang:** resources. **Yumin Luo:** resources, supervision. **Cheng Wei:** investigation. **Yongzhi Shan:** resources, supervision. **Feng Liu:** methodology. **Josemir W. Sander:** writing – review and editing, supervision. **Guoguang Zhao:** conceptualisation, funding acquisition, supervision.

## Funding

This article received support from the National Key R&D Program of China (Grant No. 2021ZD0201801), General Program of China Postdoctoral Science Foundation (Grant No. 2025M772175), and National Natural Science Foundation of China (Grant No. 82371306).

## Ethics Statement

The study used publicly available datasets, and therefore ethics approval and informed consent were not required.

## Consent

All authors have given their consent to publish.

## Conflicts of Interest

Yumin Luo is the Associate Editor of *CNS Neuroscience and Therapeutics* and a co‐author of this article. They were excluded from editorial decision‐making related to the acceptance and publication of this article. Editorial decision‐making was handled independently by the other editor to minimize bias.

## Supporting information


**Appendix S1:** Supplementary methods.
**Figure S1:** Genetic overlap between all epilepsy (EP) and subcortical volumes using MiXeR. Venn diagrams display MiXeR‐derived estimates of the number of genetic variants shared between EP and subcortical volumes. Counts of shared and unique variants for each trait are provided in thousands, including standard errors. The circle areas represent respective levels of polygenicity. L, left; R, right.
**Figure S2:** The polygenic overlap between genetic generalized epilepsy (GGE) and left thalamic volume. The conditional *Q–Q* plots reveal the cross‐trait SNP enrichment, and the negative log‐likelihood plot indicates the performance of the best model compared to the minimum and maximum models.
**Figure S3:** The polygenic overlap between genetic generalized epilepsy (GGE) and left caudate volume. The conditional *Q–Q* plots reveal the cross‐trait SNP enrichment, and the negative log‐likelihood plot indicates the performance of the best model compared to the minimum and maximum models.
**Figure S4:** The polygenic overlap between genetic generalized epilepsy (GGE) and left putamen volume. The conditional *Q–Q* plots reveal the cross‐trait SNP enrichment, and the negative log‐likelihood plot indicates the performance of the best model compared to the minimum and maximum models.
**Figure S5:** The polygenic overlap between genetic generalized epilepsy (GGE) and left pallidum volume. The conditional *Q–Q* plots reveal the cross‐trait SNP enrichment, and the negative log‐likelihood plot indicates the performance of the best model compared to the minimum and maximum models.
**Figure S6:** The polygenic overlap between genetic generalized epilepsy (GGE) and left hippocampal volume. The conditional *Q–Q* plots reveal the cross‐trait SNP enrichment, and the negative log‐likelihood plot indicates the performance of the best model compared to the minimum and maximum models.
**Figure S7:** The polygenic overlap between genetic generalized epilepsy (GGE) and left amygdala volume. The conditional *Q–Q* plots reveal the cross‐trait SNP enrichment, and the negative log‐likelihood plot indicates the performance of the best model compared to the minimum and maximum models.
**Figure S8:** The polygenic overlap between genetic generalized epilepsy (GGE) and left accumbens volume. The conditional *Q–Q* plots reveal the cross‐trait SNP enrichment, and the negative log‐likelihood plot indicates the performance of the best model compared to the minimum and maximum models.
**Figure S9:** The polygenic overlap between genetic generalized epilepsy (GGE) and left ventral diencephalon volume. The conditional *Q–Q* plots reveal the cross‐trait SNP enrichment, and the negative log‐likelihood plot indicates the performance of the best model compared to the minimum and maximum models.
**Figure S10:** The polygenic overlap between genetic generalized epilepsy (GGE) and right thalamic volume. The conditional *Q–Q* plots reveal the cross‐trait SNP enrichment, and the negative log‐likelihood plot indicates the performance of the best model compared to the minimum and maximum models.
**Figure S11:** The polygenic overlap between genetic generalized epilepsy (GGE) and right caudate volume. The conditional *Q–Q* plots reveal the cross‐trait SNP enrichment, and the negative log‐likelihood plot indicates the performance of the best model compared to the minimum and maximum models.
**Figure S12:** The polygenic overlap between genetic generalized epilepsy (GGE) and right putamen volume. The conditional *Q–Q* plots reveal the cross‐trait SNP enrichment, and the negative log‐likelihood plot indicates the performance of the best model compared to the minimum and maximum models.
**Figure S13:** The polygenic overlap between genetic generalized epilepsy (GGE) and right pallidum volume. The conditional *Q–Q* plots reveal the cross‐trait SNP enrichment, and the negative log‐likelihood plot indicates the performance of the best model compared to the minimum and maximum models.
**Figure S14:** The polygenic overlap between genetic generalized epilepsy (GGE) and right hippocampal volume. The conditional *Q–Q* plots reveal the cross‐trait SNP enrichment, and the negative log‐likelihood plot indicates the performance of the best model compared to the minimum and maximum models.
**Figure S15:** The polygenic overlap between genetic generalized epilepsy (GGE) and right amygdala volume. The conditional *Q–Q* plots reveal the cross‐trait SNP enrichment, and the negative log‐likelihood plot indicates the performance of the best model compared to the minimum and maximum models.
**Figure S16:** The polygenic overlap between genetic generalized epilepsy (GGE) and right accumbens volume. The conditional *Q–Q* plots reveal the cross‐trait SNP enrichment, and the negative log‐likelihood plot indicates the performance of the best model compared to the minimum and maximum models.
**Figure S17:** The polygenic overlap between genetic generalized epilepsy (GGE) and right ventral diencephalon volume. The conditional *Q–Q* plots reveal the cross‐trait SNP enrichment, and the negative log‐likelihood plot indicates the performance of the best model compared to the minimum and maximum models.
**Figure S18:** The polygenic overlap between all epilepsy (EP) and left thalamic volume. The conditional *Q–Q* plots reveal the cross‐trait SNP enrichment, and the negative log‐likelihood plot indicates the performance of the best model compared to the minimum and maximum models.
**Figure S19:** The polygenic overlap between all epilepsy (EP) and left caudate volume. The conditional *Q–Q* plots reveal the cross‐trait SNP enrichment, and the negative log‐likelihood plot indicates the performance of the best model compared to the minimum and maximum models.
**Figure S20:** The polygenic overlap between all epilepsy (EP) and left putamen volume. The conditional *Q–Q* plots reveal the cross‐trait SNP enrichment, and the negative log‐likelihood plot indicates the performance of the best model compared to the minimum and maximum models.
**Figure S21:** The polygenic overlap between all epilepsy (EP) and left pallidum volume. The conditional *Q–Q* plots reveal the cross‐trait SNP enrichment, and the negative log‐likelihood plot indicates the performance of the best model compared to the minimum and maximum models.
**Figure S22:** The polygenic overlap between all epilepsy (EP) and left hippocampal volume. The conditional *Q–Q* plots reveal the cross‐trait SNP enrichment, and the negative log‐likelihood plot indicates the performance of the best model compared to the minimum and maximum models.
**Figure S23:** The polygenic overlap between all epilepsy (EP) and left amygdala volume. The conditional *Q–Q* plots reveal the cross‐trait SNP enrichment, and the negative log‐likelihood plot indicates the performance of the best model compared to the minimum and maximum models.
**Figure S24:** The polygenic overlap between all epilepsy (EP) and left accumbens volume. The conditional *Q–Q* plots reveal the cross‐trait SNP enrichment, and the negative log‐likelihood plot indicates the performance of the best model compared to the minimum and maximum models.
**Figure S25:** The polygenic overlap between all epilepsy (EP) and left ventral diencephalon volume. The conditional *Q–Q* plots reveal the cross‐trait SNP enrichment, and the negative log‐likelihood plot indicates the performance of the best model compared to the minimum and maximum models.
**Figure S26:** The polygenic overlap between all epilepsy (EP) and right thalamic volume. The conditional *Q–Q* plots reveal the cross‐trait SNP enrichment, and the negative log‐likelihood plot indicates the performance of the best model compared to the minimum and maximum models.
**Figure S27:** The polygenic overlap between all epilepsy (EP) and right caudate volume. The conditional *Q–Q* plots reveal the cross‐trait SNP enrichment, and the negative log‐likelihood plot indicates the performance of the best model compared to the minimum and maximum models.
**Figure S28:** The polygenic overlap between all epilepsy (EP) and right putamen volume. The conditional *Q–Q* plots reveal the cross‐trait SNP enrichment, and the negative log‐likelihood plot indicates the performance of the best model compared to the minimum and maximum models.
**Figure S29:** The polygenic overlap between all epilepsy (EP) and right pallidum volume. The conditional *Q–Q* plots reveal the cross‐trait SNP enrichment, and the negative log‐likelihood plot indicates the performance of the best model compared to the minimum and maximum models.
**Figure S30:** The polygenic overlap between all epilepsy (EP) and right hippocampal volume. The conditional *Q–Q* plots reveal the cross‐trait SNP enrichment, and the negative log‐likelihood plot indicates the performance of the best model compared to the minimum and maximum models.
**Figure S31:** The polygenic overlap between all epilepsy (EP) and right amygdala volume. The conditional *Q–Q* plots reveal the cross‐trait SNP enrichment, and the negative log‐likelihood plot indicates the performance of the best model compared to the minimum and maximum models.
**Figure S32:** The polygenic overlap between all epilepsy (EP) and right accumbens volume. The conditional *Q–Q* plots reveal the cross‐trait SNP enrichment, and the negative log‐likelihood plot indicates the performance of the best model compared to the minimum and maximum models.
**Figure S33:** The polygenic overlap between all epilepsy (EP) and right ventral diencephalon volume. The conditional *Q–Q* plots reveal the cross‐trait SNP enrichment, and the negative log‐likelihood plot indicates the performance of the best model compared to the minimum and maximum models.
**Figure S34:** The polygenic overlap between focal epilepsy (FE) and left thalamic volume. The conditional *Q–Q* plots reveal the cross‐trait SNP enrichment, and the negative log‐likelihood plot indicates the performance of the best model compared to the minimum and maximum models.
**Figure S35:** The polygenic overlap between focal epilepsy (FE) and left caudate volume. The conditional *Q–Q* plots reveal the cross‐trait SNP enrichment, and the negative log‐likelihood plot indicates the performance of the best model compared to the minimum and maximum models.
**Figure S36:** The polygenic overlap between focal epilepsy (FE) and left putamen volume. The conditional *Q–Q* plots reveal the cross‐trait SNP enrichment, and the negative log‐likelihood plot indicates the performance of the best model compared to the minimum and maximum models.
**Figure S37:** The polygenic overlap between focal epilepsy (FE) and left pallidum volume. The conditional *Q–Q* plots reveal the cross‐trait SNP enrichment, and the negative log‐likelihood plot indicates the performance of the best model compared to the minimum and maximum models.
**Figure S38:** The polygenic overlap between focal epilepsy (FE) and left hippocampal volume. The conditional *Q–Q* plots reveal the cross‐trait SNP enrichment, and the negative log‐likelihood plot indicates the performance of the best model compared to the minimum and maximum models.
**Figure S39:** The polygenic overlap between focal epilepsy (FE) and left amygdala volume. The conditional *Q–Q* plots reveal the cross‐trait SNP enrichment, and the negative log‐likelihood plot indicates the performance of the best model compared to the minimum and maximum models.
**Figure S40:** The polygenic overlap between focal epilepsy (FE) and left accumbens volume. The conditional *Q–Q* plots reveal the cross‐trait SNP enrichment, and the negative log‐likelihood plot indicates the performance of the best model compared to the minimum and maximum models.
**Figure S41:** The polygenic overlap between focal epilepsy (FE) and left ventral diencephalon volume. The conditional *Q–Q* plots reveal the cross‐trait SNP enrichment, and the negative log‐likelihood plot indicates the performance of the best model compared to the minimum and maximum models.
**Figure S42:** The polygenic overlap between focal epilepsy (FE) and right thalamic volume. The conditional *Q–Q* plots reveal the cross‐trait SNP enrichment, and the negative log‐likelihood plot indicates the performance of the best model compared to the minimum and maximum models.
**Figure S43:** The polygenic overlap between focal epilepsy (FE) and right caudate volume. The conditional *Q–Q* plots reveal the cross‐trait SNP enrichment, and the negative log‐likelihood plot indicates the performance of the best model compared to the minimum and maximum models.
**Figure S44:** The polygenic overlap between focal epilepsy (FE) and right putamen volume. The conditional *Q–Q* plots reveal the cross‐trait SNP enrichment, and the negative log‐likelihood plot indicates the performance of the best model compared to the minimum and maximum models.
**Figure S45:** The polygenic overlap between focal epilepsy (FE) and right pallidum volume. The conditional *Q–Q* plots reveal the cross‐trait SNP enrichment, and the negative log‐likelihood plot indicates the performance of the best model compared to the minimum and maximum models.
**Figure S46:** The polygenic overlap between focal epilepsy (FE) and right hippocampal volume. The conditional *Q–Q* plots reveal the cross‐trait SNP enrichment, and the negative log‐likelihood plot indicates the performance of the best model compared to the minimum and maximum models.
**Figure S47:** The polygenic overlap between focal epilepsy (FE) and right amygdala volume. The conditional *Q–Q* plots reveal the cross‐trait SNP enrichment, and the negative log‐likelihood plot indicates the performance of the best model compared to the minimum and maximum models.
**Figure S48:** The polygenic overlap between focal epilepsy (FE) and right accumbens volume. The conditional *Q–Q* plots reveal the cross‐trait SNP enrichment, and the negative log‐likelihood plot indicates the performance of the best model compared to the minimum and maximum models.
**Figure S49:** The polygenic overlap between focal epilepsy (FE) and right ventral diencephalon volume. The conditional *Q–Q* plots reveal the cross‐trait SNP enrichment, and the negative log‐likelihood plot indicates the performance of the best model compared to the minimum and maximum models.
**Figure S50:** Manhattan plot shows the genetic associations for genetic generalized epilepsy (GGE) conditioned on each subcortical volumetric phenotype. The upper and lower panels show the results of GGE conditioned on left and right subcortical volumetric phenotypes, respectively. Individual SNPs are represented as dots throughout both panels. The most significant variants, termed lead SNPs, are distinguished by their increased size and distinctive black outlines. The vertical axis displays the negative log10‐transformed condFDR values for each genetic variant. Along the horizontal axis, SNPs are arranged according to their chromosomal positions. A horizontal dotted line marks the significance threshold at condFDR = 0.01. Brain map displaying the locus count for GGE conditioned on subcortical volumetric phenotypes. L, left; R, right.
**Figure S51:** Manhattan plot shows the genetic associations for all epilepsy (EP) conditioned on each subcortical volumetric phenotype. The upper and lower panels show the results of EP conditioned on left and right subcortical volumetric phenotypes, respectively. Individual SNPs are represented as dots throughout both panels. The most significant variants, termed lead SNPs, are distinguished by their increased size and distinctive black outlines. The vertical axis displays the negative log_10_‐transformed condFDR values for each genetic variant. Along the horizontal axis, SNPs are arranged according to their chromosomal positions. A horizontal dotted line marks the significance threshold at condFDR = 0.01. Brain map displaying the locus count for EP conditioned on subcortical volumetric phenotypes. L, left; R, right.
**Figure S52:** Manhattan plot shows the genetic associations for each subcortical volumetric phenotype conditioned on genetic generalized epilepsy (GGE). The upper and lower panels show the results of left and right subcortical volumetric phenotypes conditioned on GGE, respectively. Individual SNPs are represented as dots throughout both panels. The most significant variants, termed lead SNPs, are distinguished by their increased size and distinctive black outlines. The vertical axis displays the negative log_10_‐transformed condFDR values for each genetic variant. Along the horizontal axis, SNPs are arranged according to their chromosomal positions. A horizontal dotted line marks the significance threshold at condFDR = 0.01. Brain map displaying the locus count for each subcortical volumetric phenotype conditioned on GGE. L, left; R, right.
**Figure S53:** Manhattan plot shows the genetic associations for each subcortical volumetric phenotype conditioned on all epilepsy (EP). The upper and lower panels show the results of left and right subcortical volumetric phenotypes conditioned on EP, respectively. Individual SNPs are represented as dots throughout both panels. The most significant variants, termed lead SNPs, are distinguished by their increased size and distinctive black outlines. The vertical axis displays the negative log_10_‐transformed condFDR values for each genetic variant. Along the horizontal axis, SNPs are arranged according to their chromosomal positions. A horizontal dotted line marks the significance threshold at condFDR = 0.01. Brain map displaying the locus count for each subcortical volumetric phenotype conditioned on EP. L, left; R, right.
**Figure S54:** The number of loci identified by condFDR analysis. (A) Number and percentage of genetic generalized epilepsy (GGE) and all epilepsy (EP) loci discovered when conditioned on each subcortical volumetric phenotype. (B) Number and percentage of loci for each subcortical volumetric phenotype discovered when conditioned on GGE and EP. Bars indicate the proportion of loci in each region relative to the total loci identified across all subcortical volumetric phenotypes.
**Figure S55:** Conditional *Q–Q* plots for genetic generalized epilepsy (GGE) conditioned on left thalamic volume and vice versa.
**Figure S56:** Conditional *Q–Q* plots for genetic generalized epilepsy (GGE) conditioned on left caudate volume and vice versa.
**Figure S57:** Conditional *Q–Q* plots for genetic generalized epilepsy (GGE) conditioned on left putamen volume and vice versa.
**Figure S58:** Conditional *Q–Q* plots for genetic generalized epilepsy (GGE) conditioned on left pallidum volume and vice versa.
**Figure S59:** Conditional *Q–Q* plots for genetic generalized epilepsy (GGE) conditioned on left hippocampal volume and vice versa.
**Figure S60:** Conditional *Q–Q* plots for genetic generalized epilepsy (GGE) conditioned on left amygdala volume and vice versa.
**Figure S61:** Conditional *Q–Q* plots for genetic generalized epilepsy (GGE) conditioned on left accumbens volume and vice versa.
**Figure S62:** Conditional *Q–Q* plots for genetic generalized epilepsy (GGE) conditioned on left ventral diencephalon volume and vice versa.
**Figure S63:** Conditional *Q–Q* plots for genetic generalized epilepsy (GGE) conditioned on right thalamic volume and vice versa.
**Figure S64:** Conditional *Q–Q* plots for genetic generalized epilepsy (GGE) conditioned on right caudate volume and vice versa.
**Figure S65:** Conditional *Q–Q* plots for genetic generalized epilepsy (GGE) conditioned on right putamen volume and vice versa.
**Figure S66:** Conditional *Q–Q* plots for genetic generalized epilepsy (GGE) conditioned on right pallidum volume and vice versa.
**Figure S67:** Conditional *Q–Q* plots for genetic generalized epilepsy (GGE) conditioned on right hippocampal volume and vice versa.
**Figure S68:** Conditional *Q–Q* plots for genetic generalized epilepsy (GGE) conditioned on right amygdala volume and vice versa.
**Figure S69:** Conditional *Q–Q* plots for genetic generalized epilepsy (GGE) conditioned on right accumbens volume and vice versa.
**Figure S70:** Conditional *Q–Q* plots for genetic generalized epilepsy (GGE) conditioned on right ventral diencephalon volume and vice versa.
**Figure S71:** Conditional *Q–Q* plots for all epilepsy (EP) conditioned on left thalamic volume and vice versa.
**Figure S72:** Conditional *Q–Q* plots for all epilepsy (EP) conditioned on left caudate volume and vice versa.
**Figure S73:** Conditional *Q–Q* plots for all epilepsy (EP) conditioned on left putamen volume and vice versa.
**Figure S74:** Conditional *Q–Q* plots for all epilepsy (EP) conditioned on left pallidum volume and vice versa.
**Figure S75:** Conditional *Q–Q* plots for all epilepsy (EP) conditioned on left hippocampal volume and vice versa.
**Figure S76:** Conditional *Q–Q* plots for all epilepsy (EP) conditioned on left amygdala volume and vice versa.
**Figure S77:** Conditional *Q–Q* plots for all epilepsy (EP) conditioned on left accumbens volume and vice versa.
**Figure S78:** Conditional *Q–Q* plots for all epilepsy (EP) conditioned on left ventral diencephalon volume and vice versa.
**Figure S79:** Conditional *Q–Q* plots for all epilepsy (EP) conditioned on right thalamic volume and vice versa.
**Figure S80:** Conditional *Q–Q* plots for all epilepsy (EP) conditioned on right caudate volume and vice versa.
**Figure S81:** Conditional *Q–Q* plots for all epilepsy (EP) conditioned on right putamen volume and vice versa.
**Figure S82:** Conditional *Q–Q* plots for all epilepsy (EP) conditioned on right pallidum volume and vice versa.
**Figure S83:** Conditional *Q–Q* plots for all epilepsy (EP) conditioned on right hippocampal volume and vice versa.
**Figure S84:** Conditional *Q–Q* plots for all epilepsy (EP) conditioned on right amygdala volume and vice versa.
**Figure S85:** Conditional *Q–Q* plots for all epilepsy (EP) conditioned on right accumbens volume and vice versa.
**Figure S86:** Conditional *Q–Q* plots for all epilepsy (EP) conditioned on right ventral diencephalon volume and vice versa.
**Figure S87:** Conditional *Q–Q* plots for focal epilepsy (FE) conditioned on left thalamic volume and vice versa.
**Figure S88:** Conditional *Q–Q* plots for focal epilepsy (FE) conditioned on left caudate volume and vice versa.
**Figure S89:** Conditional *Q–Q* plots for focal epilepsy (FE) conditioned on left putamen volume and vice versa.
**Figure S90:** Conditional *Q–Q* plots for focal epilepsy (FE) conditioned on left pallidum volume and vice versa.
**Figure S91:** Conditional *Q–Q* plots for focal epilepsy (FE) conditioned on left hippocampal volume and vice versa.
**Figure S92:** Conditional *Q–Q* plots for focal epilepsy (FE) conditioned on left amygdala volume and vice versa.
**Figure S93:** Conditional *Q–Q* plots for focal epilepsy (FE) conditioned on left accumbens volume and vice versa.
**Figure S94:** Conditional *Q–Q* plots for focal epilepsy (FE) conditioned on left ventral diencephalon volume and vice versa.
**Figure S95:** Conditional *Q–Q* plots for focal epilepsy (FE) conditioned on right thalamic volume and vice versa.
**Figure S96:** Conditional *Q–Q* plots for focal epilepsy (FE) conditioned on right caudate volume and vice versa.
**Figure S97:** Conditional *Q–Q* plots for focal epilepsy (FE) conditioned on right putamen volume and vice versa.
**Figure S98:** Conditional *Q–Q* plots for focal epilepsy (FE) conditioned on right pallidum volume and vice versa.
**Figure S99:** Conditional *Q–Q* plots for focal epilepsy (FE) conditioned on right hippocampal volume and vice versa.
**Figure S100:** Conditional *Q–Q* plots for focal epilepsy (FE) conditioned on right amygdala volume and vice versa.
**Figure S101:** Conditional *Q–Q* plots for focal epilepsy (FE) conditioned on right accumbens volume and vice versa.
**Figure S102:** Conditional *Q–Q* plots for focal epilepsy (FE) conditioned on right ventral diencephalon volume and vice versa.
**Figure S103:** The Gene Ontology biological process enrichment analysis for shared genes between all epilepsy (EP) and subcortical volumes. The horizontal axis represents −log_10_‐transformed values of FDR‐corrected two‐sided *p* values, while the vertical axis displays individual Gene Ontology terms. The color intensity of each marker indicates the magnitude of −log_10_(*q*) values, and marker size corresponds to the number of genes associated with each term.
**Figure S104:** The Gene Ontology biological process enrichment analysis for genes mapped by novel genomic loci for genetic generalized epilepsy (GGE) identified by cond/conjFDR analyses. The horizontal axis represents −log_10_‐transformed values of FDR‐corrected two‐sided *p* values, while the vertical axis displays individual Gene Ontology terms. The color intensity of each marker indicates the magnitude of −log_10_(*q*) values, and marker size corresponds to the number of genes associated with each term.
**Figure S105:** The Gene Ontology biological process enrichment analysis for genes mapped by novel genomic loci for all epilepsy (EP) identified by cond/conjFDR analyses. The horizontal axis represents −log_10_‐transformed values of FDR‐corrected two‐sided *p* values, while the vertical axis displays individual Gene Ontology terms. The color intensity of each marker indicates the magnitude of −log_10_(*q*) values, and marker size corresponds to the number of genes associated with each term.

**Table S1:** Summary of previous GWAS and cond/conjFDR studies for novelty comparison of common epilepsies loci.
**Table S2:** Summary of previous GWAS and cond/conjFDR studies for novelty comparison of subcortical brain volume loci.
**Table S3:** Results of univariate MiXeR analyses.
**Table S4:** Results of bivariate MiXeR analyses between genetic generalized epilepsy (GGE) and subcortical volumes.
**Table S5:** Results of bivariate MiXeR analyses between all epilepsy (EP) and subcortical volumes.
**Table S6:** Significant loci for genetic generalized epilepsy (GGE) after conditioning on each subcortical volume.
**Table S7:** Distinct genomic loci for genetic generalized epilepsy (GGE) after conditioning on subcortical volumes.
**Table S8:** Significant loci for all epilepsy (EP) after conditioning on each subcortical volume.
**Table S9:** Distinct genomic loci for all epilepsy (EP) after conditioning on subcortical volumes.
**Table S10:** Significant loci for each subcortical volume after conditioning on genetic generalized epilepsy (GGE).
**Table S11:** Distinct genomic loci for subcortical volumes after conditioning on genetic generalized epilepsy (GGE).
**Table S12:** Significant loci for each subcortical volume after conditioning on all epilepsy (EP).
**Table S13:** Distinct genomic loci for subcortical volumes after conditioning on all epilepsy (EP).
**Table S14:** Genomic loci shared between genetic generalized epilepsy (GGE) and subcortical volumes identified by conjunctional FDR analyses.
**Table S15:** Distinct loci shared between genetic generalized epilepsy (GGE) and all subcortical volumes were identified by conjunctional FDR analyses.
**Table S16:** Genomic loci shared between all epilepsy (EP) and subcortical volumes identified by conjunctional FDR analyses.
**Table S17:** Distinct loci shared between all epilepsy (EP) and all subcortical volumes were identified by conjunctional FDR analyses.
**Table S18:** Candidate SNPs shared between genetic generalized epilepsy (GGE) and all subcortical volumes with conjunctional FDR < 0.1.
**Table S19:** Candidate SNPs shared between all epilepsy (EP) and all subcortical volumes with conjunctional FDR < 0.1.
**Table S20:** Genes mapped by candidate SNPs with conjunctional FDR < 0.1 shared between genetic generalized epilepsy (GGE) and subcortical volumes.
**Table S21:** Significant enriched biological pathways among 322 shared genes between genetic generalized epilepsy (GGE) and all subcortical volumes.
**Table S22:** Genes mapped by candidate SNPs with conjunctional FDR < 0.1 shared between all epilepsy (EP) and subcortical volumes.
**Table S23:** Significant enriched biological pathways among 159 shared genes between all epilepsy (EP) and all subcortical volumes.
**Table S24:** Genes mapped by 18 novel genomic loci for genetic generalized epilepsy (GGE) identified by conditioning on subcortical volumes.
**Table S25:** Genes mapped by 19 novel genomic loci for genetic generalized epilepsy (GGE) identified by conjunctional FDR analyses between GGE and subcortical volumes.
**Table S26:** Genes mapped by 12 novel genomic loci for all epilepsy (EP) identified by conditioning on subcortical volumes.
**Table S27:** Genes mapped by 15 novel genomic loci for all epilepsy (EP) identified by conjunctional FDR analyses between EP and subcortical volumes.
**Table S28:** Genes mapped by nine novel genomic loci for subcortical volumes identified by conditioning on genetic generalized epilepsy (GGE).
**Table S29:** Genes mapped by seven novel genomic loci for subcortical volumes identified by conditioning on all epilepsy (EP).
**Table S30:** Genes mapped by seven novel genomic loci for subcortical volumes identified by conjunctional FDR analyses between genetic generalized epilepsy (GGE) and subcortical volumes.
**Table S31:** Genes mapped by four novel genomic loci for subcortical volumes identified by conjunctional FDR analyses between all epilepsy (EP) and subcortical volumes.
**Table S32:** Significant enriched biological pathways among genes mapped by novel loci identified in genetic generalized epilepsy (GGE).
**Table S33:** Significant enriched biological pathways among genes mapped by novel loci identified in all epilepsy (EP).
**Table S34:** Associations of expression of 322 shared genes in the brain with subcortical volumes.
**Table S35:** Associations of expression of 322 shared genes in the brain with genetic generalized epilepsy (GGE).
**Table S36:** gsMap results of 49 expression‐trait associated shared genes after mapping genetic generalized epilepsy (GGE) to mouse embryonic day 16.5 spatial transcriptomics data.
**Table S37:** Details about GWAS summary for cognitive abilities, mental health and personality.
**Table S38:** Associations of expression of shared genes in the brain with cognitive abilities, mental health and personality traits.

## Data Availability

The data we used are publicly available. GWAS summary statistics of subcortical brain volumes from the UK Biobank can be accessed at https://open.win.ox.ac.uk/ukbiobank/big40/, while epilepsy GWAS summary data are retrievable from https://www.epigad.org/. The mRNA sequencing data were sourced from http://development.psychencode.org/.
