# Supplementary material for: Shared Genetic Architecture Between Common Epilepsies and Subcortical Brain Volumes Is Associated With Cognition and Mental Health
Source: CNS Neurosci Ther. 2026 Oct 5;32(10):e71156. doi: 10.1002/cns.71156 (PMC13637646; doi:10.1002/cns.71156)
Supplement: Supplementary file 1 [file CNS-32-e71156-s002.docx]

**Supplementary Information**

**Shared genetic architecture between common epilepsies and subcortical brain volumes is associated with cognition and mental health**

Zesheng Li,^1,2,3†^ Qian Wu,^4†^ Yanfeng Yang,^1,2,3†^ Jianwei Shi,^1,2,3^ Xuegang Niu,^1,2,3^ Yuanyuan Zhang,^1,2,3^ Yumin Luo,^1,2,3^ Cheng Wei,^5^ Yongzhi Shan,^1,2,3^ Feng Liu,^4^ Josemir W. Sander,^6,7^ Guoguang Zhao,^1,2,3^*

^†^ Contributed equally to this work.

**Affiliations:**

1 Department of Neurosurgery, Xuanwu Hospital, Capital Medical University, Beijing, China.

2 Clinical Research Center for Epilepsy, Capital Medical University, Beijing, China.

3 China International Neuroscience Institute, Beijing, China.

4 Department of Radiology and Tianjin Key Laboratory of Functional Imaging & Tianjin Institute of Radiology, Tianjin Medical University General Hospital, Tianjin, China.

5 Oncology and Pathology, Karolinska Institutet, 17164 Stockholm, Sweden.

6 Department of Clinical and Experimental Epilepsy, UCL Queen Square Institute of Neurology, London WC1N 3BG, & Chalfont Centre for Epilepsy, Chalfont St Peter, Buckinghamshire SL9 0RJ, UK.

7 Neurology Department, West China Hospital, Sichuan University, Chengdu 610041, China.

**Running title**: Epilepsy and subcortical volumes shared loci

**Contents**

[Supplementary Methods	8](#_Toc236641880)

[Univariate MiXeR	8](#_Toc236641881)

[Bivariate MiXeR	9](#_Toc236641882)

[Genomic locus definition	10](#_Toc236641883)

[Local genetic correlation	11](#_Toc236641884)

[Functional annotation	12](#_Toc236641885)

[Spatiotemporal gene expression trajectory analysis	13](#_Toc236641886)

[Expression-trait association analysis	14](#_Toc236641887)

[Supplementary Figures	15](#_Toc236641888)

[Supplementary Fig. 1. Genetic overlap between all epilepsy (EP) and subcortical volumes using MiXeR.	15](#_Toc236641889)

[Supplementary Fig. 2. The polygenic overlap between genetic generalized epilepsy (GGE) and left thalamic volume.	16](#_Toc236641890)

[Supplementary Fig. 3. The polygenic overlap between genetic generalized epilepsy (GGE) and left caudate volume.	16](#_Toc236641891)

[Supplementary Fig. 4. The polygenic overlap between genetic generalized epilepsy (GGE) and left putamen volume.	16](#_Toc236641892)

[Supplementary Fig. 5. The polygenic overlap between genetic generalized epilepsy (GGE) and left pallidum volume.	17](#_Toc236641893)

[Supplementary Fig. 6. The polygenic overlap between genetic generalized epilepsy (GGE) and left hippocampal volume.	17](#_Toc236641894)

[Supplementary Fig. 7. The polygenic overlap between genetic generalized epilepsy (GGE) and left amygdala volume.	17](#_Toc236641895)

[Supplementary Fig. 8. The polygenic overlap between genetic generalized epilepsy (GGE) and left accumbens volume.	18](#_Toc236641896)

[Supplementary Fig. 9. The polygenic overlap between genetic generalized epilepsy (GGE) and left ventral diencephalon volume.	18](#_Toc236641897)

[Supplementary Fig. 10. The polygenic overlap between genetic generalized epilepsy (GGE) and right thalamic volume.	18](#_Toc236641898)

[Supplementary Fig. 11. The polygenic overlap between genetic generalized epilepsy (GGE) and right caudate volume.	19](#_Toc236641899)

[Supplementary Fig. 12. The polygenic overlap between genetic generalized epilepsy (GGE) and right putamen volume.	19](#_Toc236641900)

[Supplementary Fig. 13. The polygenic overlap between genetic generalized epilepsy (GGE) and right pallidum volume.	19](#_Toc236641901)

[Supplementary Fig. 14. The polygenic overlap between genetic generalized epilepsy (GGE) and right hippocampal volume.	20](#_Toc236641902)

[Supplementary Fig. 15. The polygenic overlap between genetic generalized epilepsy (GGE) and right amygdala volume.	20](#_Toc236641903)

[Supplementary Fig. 16. The polygenic overlap between genetic generalized epilepsy (GGE) and right accumbens volume.	20](#_Toc236641904)

[Supplementary Fig. 17. The polygenic overlap between genetic generalized epilepsy (GGE) and right ventral diencephalon volume.	21](#_Toc236641905)

[Supplementary Fig. 18. The polygenic overlap between all epilepsy (EP) and left thalamic volume.	22](#_Toc236641906)

[Supplementary Fig. 19. The polygenic overlap between all epilepsy (EP) and left caudate volume.	22](#_Toc236641907)

[Supplementary Fig. 20. The polygenic overlap between all epilepsy (EP) and left putamen volume.	22](#_Toc236641908)

[Supplementary Fig. 21. The polygenic overlap between all epilepsy (EP) and left pallidum volume.	23](#_Toc236641909)

[Supplementary Fig. 22. The polygenic overlap between all epilepsy (EP) and left hippocampal volume.	23](#_Toc236641910)

[Supplementary Fig. 23. The polygenic overlap between all epilepsy (EP) and left amygdala volume.	23](#_Toc236641911)

[Supplementary Fig. 24. The polygenic overlap between all epilepsy (EP) and left accumbens volume.	24](#_Toc236641912)

[Supplementary Fig. 25. The polygenic overlap between all epilepsy (EP) and left ventral diencephalon volume.	24](#_Toc236641913)

[Supplementary Fig. 26. The polygenic overlap between all epilepsy (EP) and right thalamic volume.	24](#_Toc236641914)

[Supplementary Fig. 27. The polygenic overlap between all epilepsy (EP) and right caudate volume.	25](#_Toc236641915)

[Supplementary Fig. 28. The polygenic overlap between all epilepsy (EP) and right putamen volume.	25](#_Toc236641916)

[Supplementary Fig. 29. The polygenic overlap between all epilepsy (EP) and right pallidum volume.	25](#_Toc236641917)

[Supplementary Fig. 30. The polygenic overlap between all epilepsy (EP) and right hippocampal volume.	26](#_Toc236641918)

[Supplementary Fig. 31. The polygenic overlap between all epilepsy (EP) and right amygdala volume.	26](#_Toc236641919)

[Supplementary Fig. 32. The polygenic overlap between all epilepsy (EP) and right accumbens volume.	26](#_Toc236641920)

[Supplementary Fig. 33. The polygenic overlap between all epilepsy (EP) and right ventral diencephalon volume.	27](#_Toc236641921)

[Supplementary Fig. 34. The polygenic overlap between focal epilepsy (FE) and left thalamic volume.	28](#_Toc236641922)

[Supplementary Fig. 35. The polygenic overlap between focal epilepsy (FE) and left caudate volume.	28](#_Toc236641923)

[Supplementary Fig. 36. The polygenic overlap between focal epilepsy (FE) and left putamen volume.	28](#_Toc236641924)

[Supplementary Fig. 37. The polygenic overlap between focal epilepsy (FE) and left pallidum volume.	29](#_Toc236641925)

[Supplementary Fig. 38. The polygenic overlap between focal epilepsy (FE) and left hippocampal volume.	29](#_Toc236641926)

[Supplementary Fig. 39. The polygenic overlap between focal epilepsy (FE) and left amygdala volume.	29](#_Toc236641927)

[Supplementary Fig. 40. The polygenic overlap between focal epilepsy (FE) and left accumbens volume.	30](#_Toc236641928)

[Supplementary Fig. 41. The polygenic overlap between focal epilepsy (FE) and left ventral diencephalon volume.	30](#_Toc236641929)

[Supplementary Fig. 42. The polygenic overlap between focal epilepsy (FE) and right thalamic volume.	30](#_Toc236641930)

[Supplementary Fig. 43. The polygenic overlap between focal epilepsy (FE) and right caudate volume.	31](#_Toc236641931)

[Supplementary Fig. 44. The polygenic overlap between focal epilepsy (FE) and right putamen volume.	31](#_Toc236641932)

[Supplementary Fig. 45. The polygenic overlap between focal epilepsy (FE) and right pallidum volume.	31](#_Toc236641933)

[Supplementary Fig. 46. The polygenic overlap between focal epilepsy (FE) and right hippocampal volume.	32](#_Toc236641934)

[Supplementary Fig. 47. The polygenic overlap between focal epilepsy (FE) and right amygdala volume.	32](#_Toc236641935)

[Supplementary Fig. 48. The polygenic overlap between focal epilepsy (FE) and right accumbens volume.	32](#_Toc236641936)

[Supplementary Fig. 49. The polygenic overlap between focal epilepsy (FE) and right ventral diencephalon volume.	33](#_Toc236641937)

[Supplementary Fig. 50. Manhattan plot shows the genetic associations for genetic generalized epilepsy (GGE) conditioned on each subcortical volumetric phenotype.	34](#_Toc236641938)

[Supplementary Fig. 51. Manhattan plot shows the genetic associations for all epilepsy (EP) conditioned on each subcortical volumetric phenotype.	35](#_Toc236641939)

[Supplementary Fig. 52. Manhattan plot shows the genetic associations for each subcortical volumetric phenotype conditioned on genetic generalized epilepsy (GGE).	36](#_Toc236641940)

[Supplementary Fig. 53. Manhattan plot shows the genetic associations for each subcortical volumetric phenotype conditioned on all epilepsy (EP).	37](#_Toc236641941)

[Supplementary Fig. 54. The number of loci identified by condFDR analysis.	38](#_Toc236641942)

[Supplementary Fig. 55. Conditional *Q‒Q* plots for genetic generalized epilepsy (GGE) conditioned on left thalamic volume and vice versa.	39](#_Toc236641943)

[Supplementary Fig. 56. Conditional *Q‒Q* plots for genetic generalized epilepsy (GGE) conditioned on left caudate volume and vice versa.	39](#_Toc236641944)

[Supplementary Fig. 57. Conditional *Q‒Q* plots for genetic generalized epilepsy (GGE) conditioned on left putamen volume and vice versa.	39](#_Toc236641945)

[Supplementary Fig. 58. Conditional *Q‒Q* plots for genetic generalized epilepsy (GGE) conditioned on left pallidum volume and vice versa.	40](#_Toc236641946)

[Supplementary Fig. 59. Conditional *Q‒Q* plots for genetic generalized epilepsy (GGE) conditioned on left hippocampal volume and vice versa.	40](#_Toc236641947)

[Supplementary Fig. 60. Conditional *Q‒Q* plots for genetic generalized epilepsy (GGE) conditioned on left amygdala volume and vice versa.	40](#_Toc236641948)

[Supplementary Fig. 61. Conditional *Q‒Q* plots for genetic generalized epilepsy (GGE) conditioned on left accumbens volume and vice versa.	41](#_Toc236641949)

[Supplementary Fig. 62. Conditional *Q‒Q* plots for genetic generalized epilepsy (GGE) conditioned on left ventral diencephalon volume and vice versa.	41](#_Toc236641950)

[Supplementary Fig. 63. Conditional *Q‒Q* plots for genetic generalized epilepsy (GGE) conditioned on right thalamic volume and vice versa.	41](#_Toc236641951)

[Supplementary Fig. 64. Conditional *Q‒Q* plots for genetic generalized epilepsy (GGE) conditioned on right caudate volume and vice versa.	42](#_Toc236641952)

[Supplementary Fig. 65. Conditional *Q‒Q* plots for genetic generalized epilepsy (GGE) conditioned on right putamen volume and vice versa.	42](#_Toc236641953)

[Supplementary Fig. 66. Conditional *Q‒Q* plots for genetic generalized epilepsy (GGE) conditioned on right pallidum volume and vice versa.	42](#_Toc236641954)

[Supplementary Fig. 67. Conditional *Q‒Q* plots for genetic generalized epilepsy (GGE) conditioned on right hippocampal volume and vice versa.	43](#_Toc236641955)

[Supplementary Fig. 68. Conditional *Q‒Q* plots for genetic generalized epilepsy (GGE) conditioned on right amygdala volume and vice versa.	43](#_Toc236641956)

[Supplementary Fig. 69. Conditional *Q‒Q* plots for genetic generalized epilepsy (GGE) conditioned on right accumbens volume and vice versa.	43](#_Toc236641957)

[Supplementary Fig. 70. Conditional *Q‒Q* plots for genetic generalized epilepsy (GGE) conditioned on right ventral diencephalon volume and vice versa.	44](#_Toc236641958)

[Supplementary Fig. 71. Conditional *Q‒Q* plots for all epilepsy (EP) conditioned on left thalamic volume and vice versa.	45](#_Toc236641959)

[Supplementary Fig. 72. Conditional *Q‒Q* plots for all epilepsy (EP) conditioned on left caudate volume and vice versa.	45](#_Toc236641960)

[Supplementary Fig. 73. Conditional *Q‒Q* plots for all epilepsy (EP) conditioned on left putamen volume and vice versa.	45](#_Toc236641961)

[Supplementary Fig. 74. Conditional *Q‒Q* plots for all epilepsy (EP) conditioned on left pallidum volume and vice versa.	46](#_Toc236641962)

[Supplementary Fig. 75. Conditional *Q‒Q* plots for all epilepsy (EP) conditioned on left hippocampal volume and vice versa.	46](#_Toc236641963)

[Supplementary Fig. 76. Conditional *Q‒Q* plots for all epilepsy (EP) conditioned on left amygdala volume and vice versa.	46](#_Toc236641964)

[Supplementary Fig. 77. Conditional *Q‒Q* plots for all epilepsy (EP) conditioned on left accumbens volume and vice versa.	47](#_Toc236641965)

[Supplementary Fig. 78. Conditional *Q‒Q* plots for all epilepsy (EP) conditioned on left ventral diencephalon volume and vice versa.	47](#_Toc236641966)

[Supplementary Fig. 79. Conditional *Q‒Q* plots for all epilepsy (EP) conditioned on right thalamic volume and vice versa.	47](#_Toc236641967)

[Supplementary Fig. 80. Conditional *Q‒Q* plots for all epilepsy (EP) conditioned on right caudate volume and vice versa.	48](#_Toc236641968)

[Supplementary Fig. 81. Conditional *Q‒Q* plots for all epilepsy (EP) conditioned on right putamen volume and vice versa.	48](#_Toc236641969)

[Supplementary Fig. 82. Conditional *Q‒Q* plots for all epilepsy (EP) conditioned on right pallidum volume and vice versa.	48](#_Toc236641970)

[Supplementary Fig. 83. Conditional *Q‒Q* plots for all epilepsy (EP) conditioned on right hippocampal volume and vice versa.	49](#_Toc236641971)

[Supplementary Fig. 84. Conditional *Q‒Q* plots for all epilepsy (EP) conditioned on right amygdala volume and vice versa.	49](#_Toc236641972)

[Supplementary Fig. 85. Conditional *Q‒Q* plots for all epilepsy (EP) conditioned on right accumbens volume and vice versa.	49](#_Toc236641973)

[Supplementary Fig. 86. Conditional *Q‒Q* plots for all epilepsy (EP) conditioned on right ventral diencephalon volume and vice versa.	50](#_Toc236641974)

[Supplementary Fig. 87. Conditional *Q‒Q* plots for focal epilepsy (FE) conditioned on left thalamic volume and vice versa.	51](#_Toc236641975)

[Supplementary Fig. 88. Conditional *Q‒Q* plots for focal epilepsy (FE) conditioned on left caudate volume and vice versa.	51](#_Toc236641976)

[Supplementary Fig. 89. Conditional *Q‒Q* plots for focal epilepsy (FE) conditioned on left putamen volume and vice versa.	51](#_Toc236641977)

[Supplementary Fig. 90. Conditional *Q‒Q* plots for focal epilepsy (FE) conditioned on left pallidum volume and vice versa.	52](#_Toc236641978)

[Supplementary Fig. 91. Conditional *Q‒Q* plots for focal epilepsy (FE) conditioned on left hippocampal volume and vice versa.	52](#_Toc236641979)

[Supplementary Fig. 92. Conditional *Q‒Q* plots for focal epilepsy (FE) conditioned on left amygdala volume and vice versa.	52](#_Toc236641980)

[Supplementary Fig. 93. Conditional *Q‒Q* plots for focal epilepsy (FE) conditioned on left accumbens volume and vice versa.	53](#_Toc236641981)

[Supplementary Fig. 94. Conditional *Q‒Q* plots for focal epilepsy (FE) conditioned on left ventral diencephalon volume and vice versa.	53](#_Toc236641982)

[Supplementary Fig. 95. Conditional *Q‒Q* plots for focal epilepsy (FE) conditioned on right thalamic volume and vice versa.	53](#_Toc236641983)

[Supplementary Fig. 96. Conditional *Q‒Q* plots for focal epilepsy (FE) conditioned on right caudate volume and vice versa.	54](#_Toc236641984)

[Supplementary Fig. 97. Conditional *Q‒Q* plots for focal epilepsy (FE) conditioned on right putamen volume and vice versa.	54](#_Toc236641985)

[Supplementary Fig. 98. Conditional *Q‒Q* plots for focal epilepsy (FE) conditioned on right pallidum volume and vice versa.	54](#_Toc236641986)

[Supplementary Fig. 99. Conditional *Q‒Q* plots for focal epilepsy (FE) conditioned on right hippocampal volume and vice versa.	55](#_Toc236641987)

[Supplementary Fig. 100. Conditional *Q‒Q* plots for focal epilepsy (FE) conditioned on right amygdala volume and vice versa.	55](#_Toc236641988)

[Supplementary Fig. 101. Conditional *Q‒Q* plots for focal epilepsy (FE) conditioned on right accumbens volume and vice versa.	55](#_Toc236641989)

[Supplementary Fig. 102. Conditional *Q‒Q* plots for focal epilepsy (FE) conditioned on right ventral diencephalon volume and vice versa.	56](#_Toc236641990)

[Supplementary Fig. 103. The Gene Ontology biological process enrichment analysis for shared genes between all epilepsy (EP) and subcortical volumes.	56](#_Toc236641991)

[Supplementary Fig. 104. The Gene Ontology biological process enrichment analysis for genes mapped by novel genomic loci for genetic generalized epilepsy (GGE) identified by cond/conjFDR analyses.	56](#_Toc236641992)

[Supplementary Fig. 105. The Gene Ontology biological process enrichment analysis for genes mapped by novel genomic loci for all epilepsy (EP) identified by cond/conjFDR analyses.	57](#_Toc236641993)

# **Supplementary Methods**

## **Univariate MiXeR**

We implemented the MiXeR framework to quantify the polygenic architecture of three different types of all epilepsy (EP) and 16 brain subcortical volumes using genome-wide association study (GWAS) summary statistics. The analysis models genetic effects through a two-component Gaussian mixture distribution, integrating linkage disequilibrium (LD) and allele frequency data from the 1000 Genomes Phase 3 reference panel (n = 9,997,231 SNPs). Univariate MiXeR analyses were conducted to assess key genetic characteristics, including heritability, polygenicity, and discoverability for each phenotype. Heritability refers to the cumulative effect of all genetic variants influencing a trait, where each variant has a non-zero additive effect on the trait. Polygenicity is defined as the number of trait-influencing variants that collectively explain 90% of the heritability for each phenotype. Traits characterized by higher polygenicity are influenced by a broader range of genetic variants compared to those exhibiting lower polygenic architecture. Discoverability, defined as the variance of the effect sizes of the SNPs with non-null genetic effects, suggests that traits with higher discoverability have stronger and more detectable genetic signals.

For each SNP*i*, its standardized additive effect (*βi*) was modeled as:

$$\beta_{i}\sim\left( 1-\pi_{1} \right)\delta(0)+\pi_{1}\mathcal{N}\left( 0,\sigma_{\beta}^{2} \right)$$

where $\pi_{1}$ (“polygenicity”) is the proportion of trait-influencing ((i.e. causal SNPs et al.) SNPs and $\sigma_{\beta}^{2}$ (“discoverability”) is the variance of their effect sizes. $\delta(0)$ refers to Dirac delta function representing null effects. $\mathcal{N}\left( 0,\sigma_{\beta}^{2} \right)$ describes how the *β* values are distributed across the set of causal SNPs.

Using linkage disequilibrium (LD) and allele frequencies for SNPs from 1000 Genomes Phase 3 reference panel, Observed GWAS Z-scores ( $z_{j}$ ) were modeled as a linear combination of causal SNP effects plus residual noise:

$$z_{j}=\sum_{i} \sqrt{NH_{i}}r_{ij}\beta_{i}+\epsilon_{j}$$

Where *N* is the sample size, *Hi* is the heterozygosity of SNP *i*,$r_{ij}$ is the allelic correlation between SNPs *i* and *j*, and $\epsilon_{j}$ represents residual variance.

Parameter estimation

We estimated $\pi_{1}$, $\sigma_{\beta}^{2}$, and $\sigma_{0}^{2}$  through a computationally efficient subsampling strategy: (1) Randomly selected 2 million SNPs from the reference panel. (2) Pruned SNPs to r2<0.8*.* (3) Maximized the log-likelihood. (4) Repeated steps 1-3 across 20 independent iterations. Convergence was achieved when relative parameter changes fell below 10^−6^ or after 1,000 iterations.

The Akaike information criterion (AIC) was utilized to evaluate the performance of model fitting. Within the univariate MiXeR analysis, positive AIC values suggest that the GWAS summary statistics possess adequate statistical power to justify using the MiXeR model in comparison to the LDSC model. In the bivariate MiXeR analysis, a positive AIC value denotes that there is sufficient ability to distinguish the best-fitting model from those assuming either minimal or maximal polygenic overlap.[1]

## **Bivariate MiXeR**

Subsequently, bivariate MiXeR analyses were conducted to model the estimated additive genetic effects using a mixture of four Gaussian components. These components correspond to variants that exert no influence on either trait, variants that impact only one of the traits, and variants that affect both traits. MiXeR simultaneously estimated the proportion of shared trait-influencing variants that have concordant effects, indicating they exert the same direction of influence on both traits. To assess polygenic overlap, MiXeR computed a Dice coefficient, defined as the ratio of shared variants to the total number of variants. The results were summarized in a Venn diagram, highlighting the unique and overlapping genetic components across traits. This coefficient serves as a measure of the similarity in trait-influencing variants between epilepsy and volume traits.[2]

For cross-trait analysis, MiXeR models the joint effects (β₁ⱼ, β₂ⱼ) of SNP j as a four-component Gaussian mixture:

(𝛽_1𝑗_,𝛽_2𝑗_) ~ 𝜋_0_	δ _(0,0)_ + 𝜋_1_𝑁(0,∑_1_) + 𝜋_2_𝑁(0,∑_2_) + 𝜋_12_𝑁(0,∑_12_)

with π₀+π₁+π₂+π₁₂=1. Here π₁ and π₂ capture trait-specific causal fractions, π₁₂ the shared component, and Σ₁₂ the covariance matrix.

$$\Sigma_{12}=\left( \begin{matrix} \sigma_{1}^{2} & \rho_{12}\sigma_{1}\sigma_{2} \\ \rho_{12}\sigma_{1}\sigma_{2} & \sigma_{2}^{2} \end{matrix} \right)$$

where $\sigma_{1}^{2}$ and $\sigma_{2}^{2}$ are the univariate discoverability estimates and ρ₁₂ the correlation of shared effects. Genetic overlap was quantified by the Dice coefficient:

$$\text{Dice}=\frac{2\pi_{12}}{\pi_{1}+\pi_{2}+2\pi_{12}}$$

## **Genomic locus definition**

Independent genomic loci for the cond/conjFDR results were identified using the "sumstats.py" script, which is in line with the logic of FUMA.[3] Independent significant SNPs were identified based on thresholds of *r*²<0.6 and a condFDR<0.01 or conjFDR<0.05. Lead SNPs were identified from the significant independent variants as those with *r*²<0.1. Candidate SNPs were those with *r*²≥0.6 to any independent significant SNPs. The boundaries of each locus were established by including all such candidate SNPs. Two loci within 250 kb were merged into a single genomic locus. LD information was estimated from the 1000 Genomes European reference panel. Subsequently, independent loci across all epilepsy-volume pairs were combined into distinct loci. When the genetic positions of two or more loci from different epilepsy-volume pairs exhibited overlap, they were merged into a single distinct locus defined by the union of their genomic boundaries. For each merged locus, the lead SNP with the lowest FDR value was identified as the final lead SNP.

We determined the novelty of independent loci identified by cond/conjFDR by examining whether candidate SNPs within these loci had previously been reported in the NHGRI-EBI GWAS Catalog as significantly associated with GGE, EP, or subcortical volume phenotypes.[4] Then, our detected loci were evaluated with significant loci (p<1.0E-6) described in the original GWAS and prior cond/conjFDR work.[5–19] A locus was classified as novel if it fulfilled these criteria: (1) candidate SNPs within the locus had not been previously documented in the NHGRI-EBI GWAS Catalog; (2) the locus extended beyond 500 kb from the boundaries of any reported loci in the original GWAS summary, previous cond/conjFDR investigations, or other GWAS studies. If the locus boundaries were not provided, the genomic region was defined by creating a 2Mb window (±1000kb) centred on the lead SNP.

## **Local genetic correlation**

We employed the LAVA tool to validate the shared genetic loci identified by conjFDR analyses. The LAVA analyses were restricted to the genomic loci identified by the conjFDR framework. Since valid local genetic correlation requires sufficient local heritability, we first performed univariate filtering within LAVA. Only loci demonstrating nominally significant local genetic signal (*p*<0.05) for both phenotypes were advanced to bivariate local genetic correlation analysis. For bivariate analyses, Benjamini-Hochberg FDR *q*<0.05 is considered significant.

## **Functional annotation**

Candidate SNPs with condFDR<0.1/conjFDR<0.1 within genomic loci were functionally annotated through the FUMA platform using Combined Annotation Dependent Depletion (CADD) scores, RegulomeDB scores, and chromatin state analyses. We used CADD scores to quantify the potential deleteriousness of SNPs, with values exceeding 12.37 indicative of probable pathogenic consequences.[20] RegulomeDB scores were used to assess the regulatory functionality of SNPs, with lower scores indicating greater regulatory potential.[21] The chromatin state was used to analyse the accessibility of genomic regions, which was correlated with the transcriptional process. ChromHMM analysis across 127 epigenomic datasets generated a framework of 15 distinct chromatin states that reflect various genomic functional elements.[22] This classification integrates multiple chromatin markers to identify regulatory regions throughout the genome, including promoters, enhancers, and transcriptional activity regions. The categories (1-7) represent accessible chromatin configurations that play crucial roles in modulating gene expression and transcription.[23] Next, candidate SNPs were mapped to protein-coding genes through three approaches: (1) positional mapping of variants within a 10 kb window of gene loci, (2) cis-eQTL mapping using GTEx v8 brain data to link SNP alleles with gene expression changes, and (3) chromatin interaction mapping to capture three-dimensional DNA-DNA contacts between SNP regions and proximal or distal genes. Lastly, mapped genes were subjected to Gene Ontology (GO) biological process enrichment analysis via g:Profiler,[24] with multiple testing corrected by the Benjamini-Hochberg FDR method and terms with *q*<0.05 were deemed significant.

## **Spatiotemporal gene expression trajectory analysis**

To investigate the expression patterns of these shared genes across different lifespan stages, we analyzed processed mRNA-seq data from the PsychENCODE project.[25] This dataset includes 607 tissue samples derived from 41 postmortem brains, spanning developmental periods from 8 post-conceptional weeks (PCW) to 40 postnatal years (PY). In alignment with the methodology outlined in the PsychENCODE study,[25] the dataset consists of data collected from nine distinct developmental windows: 8-9 postconceptional weeks (PCW), 12-13 PCW, 16-17 PCW, 19-22 PCW, 35 PCW to 4 months, 0.5-2.6 PY, 2.8-10.7 PY, 13-19 PY, and 21-40 PY.[25] The gene expression data, quantified as Reads Per Kilobase of transcript per Million mapped reads (RPKM), underwent log2-transform and were subsequently normalized by centering to the mean expression level of each sample. For each volumetric trait, expression profiles were derived by aggregating values across all shared genes mapped through their shared genomic loci with epilepsy.[26] Expression trajectories were subsequently characterized using LOESS curves with statistical smoothing parameters.

## **Expression-trait association analysis**

We utilised the "S-MultiXcan" approach (<https://github.com/hakyimlab/MetaXcan>) to determine whether the expression levels of shared genes (identified by conjFDR analysis for each GGE-volume pair) correlate significantly with GGE and volumetric phenotypes.[27] This tool synthesises GWAS summary results with brain tissue-specific eQTL models derived from GTEx v8 data, covering regions such as the amygdala, anterior cingulate cortex (BA24), caudate, cerebellar hemisphere, cerebellum, cortex, frontal cortex (BA9), hippocampus, hypothalamus, nucleus accumbens, putamen, cervical spinal cord (C1), and substantia nigra. Genes with insufficient brain eQTL evidence were omitted, and statistically significant expression-trait associations were defined as Benjamini-Hochberg FDR-adjusted *q*<0.05.

# **Supplementary Figures**

**Supplementary Fig. 1. Genetic overlap between all epilepsy (EP) and subcortical volumes using MiXeR.** Venn diagrams display MiXeR-derived estimates of the number of genetic variants shared between EP and subcortical volumes. Counts of shared and unique variants for each trait are provided in thousands, including standard errors. The circle areas represent respective levels of polygenicity. L left, R right.

**Supplementary Fig. 2. The polygenic overlap between genetic generalized epilepsy (GGE) and left thalamic volume.** The conditional *Q‒Q* plots reveal the cross-trait SNP enrichment, and the negative log-likelihood plot indicates the performance of the best model compared to the minimum and maximum models.

**Supplementary Fig. 3. The polygenic overlap between genetic generalized epilepsy (GGE) and left caudate volume.** The conditional *Q‒Q* plots reveal the cross-trait SNP enrichment, and the negative log-likelihood plot indicates the performance of the best model compared to the minimum and maximum models.

**Supplementary Fig. 4. The polygenic overlap between genetic generalized epilepsy (GGE) and left putamen volume.** The conditional *Q‒Q* plots reveal the cross-trait SNP enrichment, and the negative log-likelihood plot indicates the performance of the best model compared to the minimum and maximum models.

**Supplementary Fig. 5. The polygenic overlap between genetic generalized epilepsy (GGE) and left pallidum volume.** The conditional *Q‒Q* plots reveal the cross-trait SNP enrichment, and the negative log-likelihood plot indicates the performance of the best model compared to the minimum and maximum models.

**Supplementary Fig. 6. The polygenic overlap between genetic generalized epilepsy (GGE) and left hippocampal volume.** The conditional *Q‒Q* plots reveal the cross-trait SNP enrichment, and the negative log-likelihood plot indicates the performance of the best model compared to the minimum and maximum models.

**Supplementary Fig. 7. The polygenic overlap between genetic generalized epilepsy (GGE) and left amygdala volume.** The conditional *Q‒Q* plots reveal the cross-trait SNP enrichment, and the negative log-likelihood plot indicates the performance of the best model compared to the minimum and maximum models.

**Supplementary Fig. 8. The polygenic overlap between genetic generalized epilepsy (GGE) and left accumbens volume.** The conditional *Q‒Q* plots reveal the cross-trait SNP enrichment, and the negative log-likelihood plot indicates the performance of the best model compared to the minimum and maximum models.

**Supplementary Fig. 9. The polygenic overlap between genetic generalized epilepsy (GGE) and left ventral diencephalon volume.** The conditional *Q‒Q* plots reveal the cross-trait SNP enrichment, and the negative log-likelihood plot indicates the performance of the best model compared to the minimum and maximum models.

**Supplementary Fig. 10. The polygenic overlap between genetic generalized epilepsy (GGE) and right thalamic volume.** The conditional *Q‒Q* plots reveal the cross-trait SNP enrichment, and the negative log-likelihood plot indicates the performance of the best model compared to the minimum and maximum models.

**Supplementary Fig. 11. The polygenic overlap between genetic generalized epilepsy (GGE) and right caudate volume.** The conditional *Q‒Q* plots reveal the cross-trait SNP enrichment, and the negative log-likelihood plot indicates the performance of the best model compared to the minimum and maximum models.

**Supplementary Fig. 12. The polygenic overlap between genetic generalized epilepsy (GGE) and right putamen volume.** The conditional *Q‒Q* plots reveal the cross-trait SNP enrichment, and the negative log-likelihood plot indicates the performance of the best model compared to the minimum and maximum models.

**Supplementary Fig. 13. The polygenic overlap between genetic generalized epilepsy (GGE) and right pallidum volume.** The conditional *Q‒Q* plots reveal the cross-trait SNP enrichment, and the negative log-likelihood plot indicates the performance of the best model compared to the minimum and maximum models.

**Supplementary Fig. 14. The polygenic overlap between genetic generalized epilepsy (GGE) and right hippocampal volume.** The conditional *Q‒Q* plots reveal the cross-trait SNP enrichment, and the negative log-likelihood plot indicates the performance of the best model compared to the minimum and maximum models.

**Supplementary Fig. 15. The polygenic overlap between genetic generalized epilepsy (GGE) and right amygdala volume.** The conditional *Q‒Q* plots reveal the cross-trait SNP enrichment, and the negative log-likelihood plot indicates the performance of the best model compared to the minimum and maximum models.

**Supplementary Fig. 16. The polygenic overlap between genetic generalized epilepsy (GGE) and right accumbens volume.** The conditional *Q‒Q* plots reveal the cross-trait SNP enrichment, and the negative log-likelihood plot indicates the performance of the best model compared to the minimum and maximum models.

**Supplementary Fig. 17. The polygenic overlap between genetic generalized epilepsy (GGE) and right ventral diencephalon volume.** The conditional *Q‒Q* plots reveal the cross-trait SNP enrichment, and the negative log-likelihood plot indicates the performance of the best model compared to the minimum and maximum models.

**Supplementary Fig. 18. The polygenic overlap between all epilepsy (EP) and left thalamic volume.** The conditional *Q‒Q* plots reveal the cross-trait SNP enrichment, and the negative log-likelihood plot indicates the performance of the best model compared to the minimum and maximum models.

**Supplementary Fig. 19. The polygenic overlap between all epilepsy (EP) and left caudate volume.** The conditional *Q‒Q* plots reveal the cross-trait SNP enrichment, and the negative log-likelihood plot indicates the performance of the best model compared to the minimum and maximum models.

**Supplementary Fig. 20. The polygenic overlap between all epilepsy (EP) and left putamen volume.** The conditional *Q‒Q* plots reveal the cross-trait SNP enrichment, and the negative log-likelihood plot indicates the performance of the best model compared to the minimum and maximum models.

**Supplementary Fig. 21. The polygenic overlap between all epilepsy (EP) and left pallidum volume.** The conditional *Q‒Q* plots reveal the cross-trait SNP enrichment, and the negative log-likelihood plot indicates the performance of the best model compared to the minimum and maximum models.

**Supplementary Fig. 22. The polygenic overlap between all epilepsy (EP) and left hippocampal volume.** The conditional *Q‒Q* plots reveal the cross-trait SNP enrichment, and the negative log-likelihood plot indicates the performance of the best model compared to the minimum and maximum models.

**Supplementary Fig. 23. The polygenic overlap between all epilepsy (EP) and left amygdala volume.** The conditional *Q‒Q* plots reveal the cross-trait SNP enrichment, and the negative log-likelihood plot indicates the performance of the best model compared to the minimum and maximum models.

**Supplementary Fig. 24. The polygenic overlap between all epilepsy (EP) and left accumbens volume.** The conditional *Q‒Q* plots reveal the cross-trait SNP enrichment, and the negative log-likelihood plot indicates the performance of the best model compared to the minimum and maximum models.

**Supplementary Fig. 25. The polygenic overlap between all epilepsy (EP) and left ventral diencephalon volume.** The conditional *Q‒Q* plots reveal the cross-trait SNP enrichment, and the negative log-likelihood plot indicates the performance of the best model compared to the minimum and maximum models.

**Supplementary Fig. 26. The polygenic overlap between all epilepsy (EP) and right thalamic volume.** The conditional *Q‒Q* plots reveal the cross-trait SNP enrichment, and the negative log-likelihood plot indicates the performance of the best model compared to the minimum and maximum models.

**Supplementary Fig. 27. The polygenic overlap between all epilepsy (EP) and right caudate volume.** The conditional *Q‒Q* plots reveal the cross-trait SNP enrichment, and the negative log-likelihood plot indicates the performance of the best model compared to the minimum and maximum models.

**Supplementary Fig. 28. The polygenic overlap between all epilepsy (EP) and right putamen volume.** The conditional *Q‒Q* plots reveal the cross-trait SNP enrichment, and the negative log-likelihood plot indicates the performance of the best model compared to the minimum and maximum models.

**Supplementary Fig. 29. The polygenic overlap between all epilepsy (EP) and right pallidum volume.** The conditional *Q‒Q* plots reveal the cross-trait SNP enrichment, and the negative log-likelihood plot indicates the performance of the best model compared to the minimum and maximum models.

**Supplementary Fig. 30. The polygenic overlap between all epilepsy (EP) and right hippocampal volume.** The conditional *Q‒Q* plots reveal the cross-trait SNP enrichment, and the negative log-likelihood plot indicates the performance of the best model compared to the minimum and maximum models.

**Supplementary Fig. 31. The polygenic overlap between all epilepsy (EP) and right amygdala volume.** The conditional *Q‒Q* plots reveal the cross-trait SNP enrichment, and the negative log-likelihood plot indicates the performance of the best model compared to the minimum and maximum models.

**Supplementary Fig. 32. The polygenic overlap between all epilepsy (EP) and right accumbens volume.** The conditional *Q‒Q* plots reveal the cross-trait SNP enrichment, and the negative log-likelihood plot indicates the performance of the best model compared to the minimum and maximum models.

**Supplementary Fig. 33. The polygenic overlap between all epilepsy (EP) and right ventral diencephalon volume.** The conditional *Q‒Q* plots reveal the cross-trait SNP enrichment, and the negative log-likelihood plot indicates the performance of the best model compared to the minimum and maximum models.

**Supplementary Fig. 34. The polygenic overlap between focal epilepsy (FE) and left thalamic volume.** The conditional *Q‒Q* plots reveal the cross-trait SNP enrichment, and the negative log-likelihood plot indicates the performance of the best model compared to the minimum and maximum models.

**Supplementary Fig. 35. The polygenic overlap between focal epilepsy (FE) and left caudate volume.** The conditional *Q‒Q* plots reveal the cross-trait SNP enrichment, and the negative log-likelihood plot indicates the performance of the best model compared to the minimum and maximum models.

**Supplementary Fig. 36. The polygenic overlap between focal epilepsy (FE) and left putamen volume.** The conditional *Q‒Q* plots reveal the cross-trait SNP enrichment, and the negative log-likelihood plot indicates the performance of the best model compared to the minimum and maximum models.

**Supplementary Fig. 37. The polygenic overlap between focal epilepsy (FE) and left pallidum volume.** The conditional *Q‒Q* plots reveal the cross-trait SNP enrichment, and the negative log-likelihood plot indicates the performance of the best model compared to the minimum and maximum models.

**Supplementary Fig. 38. The polygenic overlap between focal epilepsy (FE) and left hippocampal volume.** The conditional *Q‒Q* plots reveal the cross-trait SNP enrichment, and the negative log-likelihood plot indicates the performance of the best model compared to the minimum and maximum models.

**Supplementary Fig. 39. The polygenic overlap between focal epilepsy (FE) and left amygdala volume.** The conditional *Q‒Q* plots reveal the cross-trait SNP enrichment, and the negative log-likelihood plot indicates the performance of the best model compared to the minimum and maximum models.

**Supplementary Fig. 40. The polygenic overlap between focal epilepsy (FE) and left accumbens volume.** The conditional *Q‒Q* plots reveal the cross-trait SNP enrichment, and the negative log-likelihood plot indicates the performance of the best model compared to the minimum and maximum models.

**Supplementary Fig. 41. The polygenic overlap between focal epilepsy (FE) and left ventral diencephalon volume.** The conditional *Q‒Q* plots reveal the cross-trait SNP enrichment, and the negative log-likelihood plot indicates the performance of the best model compared to the minimum and maximum models.

**Supplementary Fig. 42. The polygenic overlap between focal epilepsy (FE) and right thalamic volume.** The conditional *Q‒Q* plots reveal the cross-trait SNP enrichment, and the negative log-likelihood plot indicates the performance of the best model compared to the minimum and maximum models.

**Supplementary Fig. 43. The polygenic overlap between focal epilepsy (FE) and right caudate volume.** The conditional *Q‒Q* plots reveal the cross-trait SNP enrichment, and the negative log-likelihood plot indicates the performance of the best model compared to the minimum and maximum models.

**Supplementary Fig. 44. The polygenic overlap between focal epilepsy (FE) and right putamen volume.** The conditional *Q‒Q* plots reveal the cross-trait SNP enrichment, and the negative log-likelihood plot indicates the performance of the best model compared to the minimum and maximum models.

**Supplementary Fig. 45. The polygenic overlap between focal epilepsy (FE) and right pallidum volume.** The conditional *Q‒Q* plots reveal the cross-trait SNP enrichment, and the negative log-likelihood plot indicates the performance of the best model compared to the minimum and maximum models.

**Supplementary Fig. 46. The polygenic overlap between focal epilepsy (FE) and right hippocampal volume.** The conditional *Q‒Q* plots reveal the cross-trait SNP enrichment, and the negative log-likelihood plot indicates the performance of the best model compared to the minimum and maximum models.

**Supplementary Fig. 47. The polygenic overlap between focal epilepsy (FE) and right amygdala volume.** The conditional *Q‒Q* plots reveal the cross-trait SNP enrichment, and the negative log-likelihood plot indicates the performance of the best model compared to the minimum and maximum models.

**Supplementary Fig. 48. The polygenic overlap between focal epilepsy (FE) and right accumbens volume.** The conditional *Q‒Q* plots reveal the cross-trait SNP enrichment, and the negative log-likelihood plot indicates the performance of the best model compared to the minimum and maximum models.

**Supplementary Fig. 49. The polygenic overlap between focal epilepsy (FE) and right ventral diencephalon volume.** The conditional *Q‒Q* plots reveal the cross-trait SNP enrichment, and the negative log-likelihood plot indicates the performance of the best model compared to the minimum and maximum models.

**Supplementary Fig. 50. Manhattan plot shows the genetic associations for genetic generalized epilepsy (GGE) conditioned on each subcortical volumetric phenotype.** The upper and lower panels show the results of GGE conditioned on left and right subcortical volumetric phenotypes, respectively. Individual SNPs are represented as dots throughout both panels. The most significant variants, termed lead SNPs, are distinguished by their increased size and distinctive black outlines. The vertical axis displays the negative log10-transformed condFDR values for each genetic variant. Along the horizontal axis, SNPs are arranged according to their chromosomal positions. A horizontal dotted line marks the significance threshold at condFDR = 0.01. Brain map displaying the locus count for GGE conditioned on subcortical volumetric phenotypes. L left, R right.

**Supplementary Fig. 51. Manhattan plot shows the genetic associations for all epilepsy (EP) conditioned on each subcortical volumetric phenotype.** The upper and lower panels show the results of EP conditioned on left and right subcortical volumetric phenotypes, respectively. Individual SNPs are represented as dots throughout both panels. The most significant variants, termed lead SNPs, are distinguished by their increased size and distinctive black outlines. The vertical axis displays the negative log10-transformed condFDR values for each genetic variant. Along the horizontal axis, SNPs are arranged according to their chromosomal positions. A horizontal dotted line marks the significance threshold at condFDR = 0.01. Brain map displaying the locus count for EP conditioned on subcortical volumetric phenotypes. L left, R right.

.

**Supplementary Fig. 52. Manhattan plot shows the genetic associations for each subcortical volumetric phenotype conditioned on genetic generalized epilepsy (GGE).** The upper and lower panels show the results of left and right subcortical volumetric phenotypes conditioned on GGE, respectively. Individual SNPs are represented as dots throughout both panels. The most significant variants, termed lead SNPs, are distinguished by their increased size and distinctive black outlines. The vertical axis displays the negative log10-transformed condFDR values for each genetic variant. Along the horizontal axis, SNPs are arranged according to their chromosomal positions. A horizontal dotted line marks the significance threshold at condFDR = 0.01. Brain map displaying the locus count for each subcortical volumetric phenotype conditioned on GGE. L left, R right.

**Supplementary Fig. 53. Manhattan plot shows the genetic associations for each subcortical volumetric phenotype conditioned on all epilepsy (EP).** The upper and lower panels show the results of left and right subcortical volumetric phenotypes conditioned on EP, respectively. Individual SNPs are represented as dots throughout both panels. The most significant variants, termed lead SNPs, are distinguished by their increased size and distinctive black outlines. The vertical axis displays the negative log10-transformed condFDR values for each genetic variant. Along the horizontal axis, SNPs are arranged according to their chromosomal positions. A horizontal dotted line marks the significance threshold at condFDR = 0.01. Brain map displaying the locus count for each subcortical volumetric phenotype conditioned on EP. L left, R right.

**Supplementary Fig. 54. The number of loci identified by condFDR analysis.** A. Number and percentage of genetic generalized epilepsy (GGE) and all epilepsy (EP) loci discovered when conditioned on each subcortical volumetric phenotype. B. Number and percentage of loci for each subcortical volumetric phenotype discovered when conditioned on GGE and EP. Bars indicate the proportion of loci in each region relative to the total loci identified across all subcortical volumetric phenotypes.

## **Supplementary Fig. 55. Conditional *Q‒Q* plots for genetic generalized epilepsy (GGE) conditioned on left thalamic volume and vice versa.**

## **Supplementary Fig. 56. Conditional *Q‒Q* plots for genetic generalized epilepsy (GGE) conditioned on left caudate volume and vice versa.**

## **Supplementary Fig. 57. Conditional *Q‒Q* plots for genetic generalized epilepsy (GGE) conditioned on left putamen volume and vice versa.**

## **Supplementary Fig. 58. Conditional *Q‒Q* plots for genetic generalized epilepsy (GGE) conditioned on left pallidum volume and vice versa.**

## **Supplementary Fig. 59. Conditional *Q‒Q* plots for genetic generalized epilepsy (GGE) conditioned on left hippocampal volume and vice versa.**

## **Supplementary Fig. 60. Conditional *Q‒Q* plots for genetic generalized epilepsy (GGE) conditioned on left amygdala volume and vice versa.**

## **Supplementary Fig. 61. Conditional *Q‒Q* plots for genetic generalized epilepsy (GGE) conditioned on left accumbens volume and vice versa.**

## **Supplementary Fig. 62. Conditional *Q‒Q* plots for genetic generalized epilepsy (GGE) conditioned on left ventral diencephalon volume and vice versa.**

## **Supplementary Fig. 63. Conditional *Q‒Q* plots for genetic generalized epilepsy (GGE) conditioned on right thalamic volume and vice versa.**

## **Supplementary Fig. 64. Conditional *Q‒Q* plots for genetic generalized epilepsy (GGE) conditioned on right caudate volume and vice versa.**

## **Supplementary Fig. 65. Conditional *Q‒Q* plots for genetic generalized epilepsy (GGE) conditioned on right putamen volume and vice versa.**

## **Supplementary Fig. 66. Conditional *Q‒Q* plots for genetic generalized epilepsy (GGE) conditioned on right pallidum volume and vice versa.**

## **Supplementary Fig. 67. Conditional *Q‒Q* plots for genetic generalized epilepsy (GGE) conditioned on right hippocampal volume and vice versa.**

## **Supplementary Fig. 68. Conditional *Q‒Q* plots for genetic generalized epilepsy (GGE) conditioned on right amygdala volume and vice versa.**

## **Supplementary Fig. 69. Conditional *Q‒Q* plots for genetic generalized epilepsy (GGE) conditioned on right accumbens volume and vice versa.**

## **Supplementary Fig. 70. Conditional *Q‒Q* plots for genetic generalized epilepsy (GGE) conditioned on right ventral diencephalon volume and vice versa.**

## **Supplementary Fig. 71. Conditional *Q‒Q* plots for all epilepsy (EP) conditioned on left thalamic volume and vice versa.**

## **Supplementary Fig. 72. Conditional *Q‒Q* plots for all epilepsy (EP) conditioned on left caudate volume and vice versa.**

## **Supplementary Fig. 73. Conditional *Q‒Q* plots for all epilepsy (EP) conditioned on left putamen volume and vice versa.**

## **Supplementary Fig. 74. Conditional *Q‒Q* plots for all epilepsy (EP) conditioned on left pallidum volume and vice versa.**

## **Supplementary Fig. 75. Conditional *Q‒Q* plots for all epilepsy (EP) conditioned on left hippocampal volume and vice versa.**

## **Supplementary Fig. 76. Conditional *Q‒Q* plots for all epilepsy (EP) conditioned on left amygdala volume and vice versa.**

## **Supplementary Fig. 77. Conditional *Q‒Q* plots for all epilepsy (EP) conditioned on left accumbens volume and vice versa.**

## **Supplementary Fig. 78. Conditional *Q‒Q* plots for all epilepsy (EP) conditioned on left ventral diencephalon volume and vice versa.**

## **Supplementary Fig. 79. Conditional *Q‒Q* plots for all epilepsy (EP) conditioned on right thalamic volume and vice versa.**

## **Supplementary Fig. 80. Conditional *Q‒Q* plots for all epilepsy (EP) conditioned on right caudate volume and vice versa.**

## **Supplementary Fig. 81. Conditional *Q‒Q* plots for all epilepsy (EP) conditioned on right putamen volume and vice versa.**

## **Supplementary Fig. 82. Conditional *Q‒Q* plots for all epilepsy (EP) conditioned on right pallidum volume and vice versa.**

## **Supplementary Fig. 83. Conditional *Q‒Q* plots for all epilepsy (EP) conditioned on right hippocampal volume and vice versa.**

## **Supplementary Fig. 84. Conditional *Q‒Q* plots for all epilepsy (EP) conditioned on right amygdala volume and vice versa.**

## **Supplementary Fig. 85. Conditional *Q‒Q* plots for all epilepsy (EP) conditioned on right accumbens volume and vice versa.**

## **Supplementary Fig. 86. Conditional *Q‒Q* plots for all epilepsy (EP) conditioned on right ventral diencephalon volume and vice versa.**

## **Supplementary Fig. 87. Conditional *Q‒Q* plots for focal epilepsy (FE) conditioned on left thalamic volume and vice versa.**

## **Supplementary Fig. 88. Conditional *Q‒Q* plots for focal epilepsy (FE) conditioned on left caudate volume and vice versa.**

## **Supplementary Fig. 89. Conditional *Q‒Q* plots for focal epilepsy (FE) conditioned on left putamen volume and vice versa.**

## **Supplementary Fig. 90. Conditional *Q‒Q* plots for focal epilepsy (FE) conditioned on left pallidum volume and vice versa.**

## **Supplementary Fig. 91. Conditional *Q‒Q* plots for focal epilepsy (FE) conditioned on left hippocampal volume and vice versa.**

## **Supplementary Fig. 92. Conditional *Q‒Q* plots for focal epilepsy (FE) conditioned on left amygdala volume and vice versa.**

## **Supplementary Fig. 93. Conditional *Q‒Q* plots for focal epilepsy (FE) conditioned on left accumbens volume and vice versa.**

## **Supplementary Fig. 94. Conditional *Q‒Q* plots for focal epilepsy (FE) conditioned on left ventral diencephalon volume and vice versa.**

## **Supplementary Fig. 95. Conditional *Q‒Q* plots for focal epilepsy (FE) conditioned on right thalamic volume and vice versa.**

## **Supplementary Fig. 96. Conditional *Q‒Q* plots for focal epilepsy (FE) conditioned on right caudate volume and vice versa.**

## **Supplementary Fig. 97. Conditional *Q‒Q* plots for focal epilepsy (FE) conditioned on right putamen volume and vice versa.**

## **Supplementary Fig. 98. Conditional *Q‒Q* plots for focal epilepsy (FE) conditioned on right pallidum volume and vice versa.**

## **Supplementary Fig. 99. Conditional *Q‒Q* plots for focal epilepsy (FE) conditioned on right hippocampal volume and vice versa.**

## **Supplementary Fig. 100. Conditional *Q‒Q* plots for focal epilepsy (FE) conditioned on right amygdala volume and vice versa.**

## **Supplementary Fig. 101. Conditional *Q‒Q* plots for focal epilepsy (FE) conditioned on right accumbens volume and vice versa.**

## **Supplementary Fig. 102. Conditional *Q‒Q* plots for focal epilepsy (FE) conditioned on right ventral diencephalon volume and vice versa.**

## **Supplementary Fig. 103. The Gene Ontology biological process enrichment analysis for shared genes between all epilepsy (EP) and subcortical volumes.** The horizontal axis represents -log10-transformed values of FDR-corrected two-sided *p*-values, while the vertical axis displays individual Gene Ontology terms. The color intensity of each marker indicates the magnitude of -log10(*q*) values, and marker size corresponds to the number of genes associated with each term.

## **Supplementary Fig. 104. The Gene Ontology biological process enrichment analysis for genes mapped by novel genomic loci for genetic generalized epilepsy (GGE) identified by cond/conjFDR analyses.** The horizontal axis represents -log10-transformed values of FDR-corrected two-sided *p*-values, while the vertical axis displays individual Gene Ontology terms. The color intensity of each marker indicates the magnitude of -log10(*q*) values, and marker size corresponds to the number of genes associated with each term.

## **Supplementary Fig. 105. The Gene Ontology biological process enrichment analysis for genes mapped by novel genomic loci for all epilepsy (EP) identified by cond/conjFDR analyses.** The horizontal axis represents -log10-transformed values of FDR-corrected two-sided *p*-values, while the vertical axis displays individual Gene Ontology terms. The color intensity of each marker indicates the magnitude of -log10(*q*) values, and marker size corresponds to the number of genes associated with each term.

**Reference**

1. Holland D, Frei O, Desikan R, Fan C-C, Shadrin AA, Smeland OB, et al. Beyond SNP heritability: Polygenicity and discoverability of phenotypes estimated with a univariate Gaussian mixture model. PLoS Genet. 2020;16:e1008612. https://doi.org/10.1371/journal.pgen.1008612

2. Frei O, Holland D, Smeland OB, Shadrin AA, Fan CC, Maeland S, et al. Bivariate causal mixture model quantifies polygenic overlap between complex traits beyond genetic correlation. Nat Commun. 2019;10:2417. https://doi.org/10.1038/s41467-019-10310-0

3. Watanabe K, Taskesen E, van Bochoven A, Posthuma D. Functional mapping and annotation of genetic associations with FUMA. Nat Commun. 2017;8:1826. https://doi.org/10.1038/s41467-017-01261-5

4. Buniello A, MacArthur JAL, Cerezo M, Harris LW, Hayhurst J, Malangone C, et al. The NHGRI-EBI GWAS Catalog of published genome-wide association studies, targeted arrays and summary statistics 2019. Nucleic Acids Res. 2019;47:D1005–12. https://doi.org/10.1093/nar/gky1120

5. Karadag N, Shadrin AA, O’Connell KS, Hindley GFL, Rahman Z, Parker N, et al. Identification of novel genomic risk loci shared between common epilepsies and psychiatric disorders. Brain. 2023;146:3392–403. https://doi.org/10.1093/brain/awad038

6. Hibar DP, Stein JL, Renteria ME, Arias-Vasquez A, Desrivières S, Jahanshad N, et al. Common genetic variants influence human subcortical brain structures. Nature. 2015;520:224–9. https://doi.org/10.1038/nature14101

7. International League Against Epilepsy Consortium on Complex Epilepsies. GWAS meta-analysis of over 29,000 people with epilepsy identifies 26 risk loci and subtype-specific genetic architecture. Nat Genet. 2023;55:1471–82. https://doi.org/10.1038/s41588-023-01485-w

8. International League Against Epilepsy Consortium on Complex Epilepsies. Genome-wide mega-analysis identifies 16 loci and highlights diverse biological mechanisms in the common epilepsies. Nat Commun. 2018;9:5269. https://doi.org/10.1038/s41467-018-07524-z

9. García-Marín LM, Campos AI, Diaz-Torres S, Rabinowitz JA, Ceja Z, Mitchell BL, et al. Genomic analysis of intracranial and subcortical brain volumes yields polygenic scores accounting for variation across ancestries. Nat Genet. 2024;56:2333–44. https://doi.org/10.1038/s41588-024-01951-z

10. Zhao Q, Xu J, Shi Z, Zhang Y, Du X, Zhai Y, et al. Genome-wide Pleiotropy Analysis Reveals Shared Genetic Associations between Type 2 Diabetes Mellitus and Subcortical Brain Volumes. Research (Wash D C). 2025;8:0688. https://doi.org/10.34133/research.0688

11. Liu M, Wang L, Zhang Y, Dong H, Wang C, Chen Y, et al. Investigating the shared genetic architecture between depression and subcortical volumes. Nat Commun. 2024;15:7647. https://doi.org/10.1038/s41467-024-52121-y

12. Bahrami S, Nordengen K, Rokicki J, Shadrin AA, Rahman Z, Smeland OB, et al. The genetic landscape of basal ganglia and implications for common brain disorders. Nat Commun. 2024;15:8476. https://doi.org/10.1038/s41467-024-52583-0

13. Cheng W, van der Meer D, Parker N, Hindley G, O’Connell KS, Wang Y, et al. Shared genetic architecture between schizophrenia and subcortical brain volumes implicates early neurodevelopmental processes and brain development in childhood. Mol Psychiatry. 2022;27:5167–76. https://doi.org/10.1038/s41380-022-01751-z

14. Elvsåshagen T, Shadrin A, Frei O, van der Meer D, Bahrami S, Kumar VJ, et al. The genetic architecture of the human thalamus and its overlap with ten common brain disorders. Nat Commun. 2021;12:2909. https://doi.org/10.1038/s41467-021-23175-z

15. Satizabal CL, Adams HHH, Hibar DP, White CC, Knol MJ, Stein JL, et al. Genetic architecture of subcortical brain structures in 38,851 individuals. Nat Genet. 2019;51:1624–36. https://doi.org/10.1038/s41588-019-0511-y

16. Karadag N, Hagen E, Shadrin AA, van der Meer D, O’Connell KS, Rahman Z, et al. Unraveling the shared genetics of common epilepsies and general cognitive ability. Seizure. 2024;122:105–12. https://doi.org/10.1016/j.seizure.2024.09.016

17. International League Against Epilepsy Consortium on Complex Epilepsies. Electronic address: epilepsy-austin@unimelb.edu.au. Genetic determinants of common epilepsies: a meta-analysis of genome-wide association studies. Lancet Neurol. 2014;13:893–903. https://doi.org/10.1016/S1474-4422(14)70171-1

18. EPICURE Consortium, EMINet Consortium, Steffens M, Leu C, Ruppert A-K, Zara F, et al. Genome-wide association analysis of genetic generalized epilepsies implicates susceptibility loci at 1q43, 2p16.1, 2q22.3 and 17q21.32. Hum Mol Genet. 2012;21:5359–72. https://doi.org/10.1093/hmg/dds373

19. Wu Y, Li Y, Zhu J, Long J. Shared genetics and causality underlying epilepsy and attention-deficit hyperactivity disorder. Psychiatry Res. 2022;316:114794. https://doi.org/10.1016/j.psychres.2022.114794

20. Kircher M, Witten DM, Jain P, O’Roak BJ, Cooper GM, Shendure J. A general framework for estimating the relative pathogenicity of human genetic variants. Nat Genet. 2014;46:310–5. https://doi.org/10.1038/ng.2892

21. Boyle AP, Hong EL, Hariharan M, Cheng Y, Schaub MA, Kasowski M, et al. Annotation of functional variation in personal genomes using RegulomeDB. Genome Res. 2012;22:1790–7. https://doi.org/10.1101/gr.137323.112

22. Ernst J, Kellis M. ChromHMM: automating chromatin-state discovery and characterization. Nat Methods. 2012;9:215–6. https://doi.org/10.1038/nmeth.1906

23. Roadmap Epigenomics Consortium, Kundaje A, Meuleman W, Ernst J, Bilenky M, Yen A, et al. Integrative analysis of 111 reference human epigenomes. Nature. 2015;518:317–30. https://doi.org/10.1038/nature14248

24. Kolberg L, Raudvere U, Kuzmin I, Adler P, Vilo J, Peterson H. g:Profiler-interoperable web service for functional enrichment analysis and gene identifier mapping (2023 update). Nucleic Acids Res. 2023;51:W207–12. https://doi.org/10.1093/nar/gkad347

25. Li M, Santpere G, Imamura Kawasawa Y, Evgrafov OV, Gulden FO, Pochareddy S, et al. Integrative functional genomic analysis of human brain development and neuropsychiatric risks. Science. 2018;362:eaat7615. https://doi.org/10.1126/science.aat7615

26. Cross-Disorder Group of the Psychiatric Genomics Consortium. Electronic address: plee0@mgh.harvard.edu, Cross-Disorder Group of the Psychiatric Genomics Consortium. Genomic Relationships, Novel Loci, and Pleiotropic Mechanisms across Eight Psychiatric Disorders. Cell. 2019;179:1469-1482.e11. https://doi.org/10.1016/j.cell.2019.11.020

27. Barbeira AN, Pividori M, Zheng J, Wheeler HE, Nicolae DL, Im HK. Integrating predicted transcriptome from multiple tissues improves association detection. PLoS Genet. 2019;15:e1007889. https://doi.org/10.1371/journal.pgen.1007889
